# Heterogeneous foraging swarms can be better

**DOI:** 10.3389/frobt.2024.1426282

**Published:** 2025-01-20

**Authors:** Gal A. Kaminka, Yinon Douchan

**Affiliations:** Department of Computer Science, Gonda Brain Research Center, and Nanotechnology Center, Bar Ilan University, Ramat Gan, Israel

**Keywords:** multi-agent reinforcement learning, foraging, swarm robotics, heterogeneous robots, robot diversity, difference reward, marginal contribution, game theory

## Abstract

**Introduction:**

Inspired by natural phenomena, generations of researchers have been investigating how a swarm of robots can act coherently and purposefully, when individual robots can only sense and communicate with nearby peers, with no means of global communications and coordination. In this paper, we will show that swarms can perform better, when they self-adapt to admit heterogeneous behavior roles.

**Methods:**

We model a foraging swarm task as an extensive-form fully-cooperative game, in which the swarm reward is an additive function of individual contributions (the sum of collected items). To maximize the swarm reward, previous work proposed using distributed reinforcement learning, where each robot adapts its own collision-avoidance decisions based on the Effectiveness Index reward (*EI*). *EI* uses information about the time between their own collisions (information readily available even to simple physical robots). While promising, the use of *EI* is brittle (as we show), since robots that selfishly seek to optimize their own *EI* (minimizing time spent on collisions) can actually cause swarm-wide performance to degrade.

**Results:**

To address this, we derive a reward function from a game-theoretic view of swarm foraging as a fully-cooperative, unknown horizon repeating game. We demonstrate analytically that the total coordination overhead of the swarm (total time spent on collision-avoidance, rather than foraging per-se) is directly tied to the total utility of the swarm: less overhead, more items collected. Treating every collision as a stage in the repeating game, the overhead is bounded by the total *EI* of all robots. We then use a marginal-contribution (difference-reward) formulation to derive individual rewards from the total *EI*. The resulting Aligned Effective Index (AEI) reward has the property that each individual can estimate the impact of its decisions on the swarm: individual improvements translate to swarm improvements. We show that AEI provably generalizes previous work, adding a component that computes the effect of counterfactual robot absence. Different assumptions on this counterfactual lead to bounds on AEI from above and below.

**Discussion:**

While the theoretical analysis clarifies both assumptions and gaps with respect to the reality of robots, experiments with real and simulated robots empirically demonstrate the efficacy of the approach in practice, and the importance of behavioral (decision-making) diversity in optimizing swarm goals.

## 1 Introduction

Distributed multi-robot systems comprise multiple robots, each under its own control (Farinelli et al., 2004; Parker, 2008). Typically, the robots are deployed to carry out tasks toward a global goal. Examples include coverage (Agmon et al., 2008a; Hazon and Kaminka, 2008; Yehoshua et al., 2016; Giuggioli et al., 2016; Rekleitis et al., 2008); patrolling (Sempe and Drogoul, 2003; Elmaliach et al., 2007; Elmaliach et al., 2008; Agmon et al., 2008b; Elmaliach et al., 2009; Basilico et al., 2009; Marino et al., 2009; Jensen et al., 2011; Portugal and Rocha, 2013; Yan and Zhang, 2016); formation maintenance (Kaminka and Glick, 2006; Kaminka et al., 2008; Michaud et al., 2002; Fredslund and Mataric, 2002; Desai, 2002; Desai et al., 1998; Kaminka et al., 2013; 2016; Balch and Arkin, 1998; Lemay et al., 2004; Michael et al., 2008); multi-agent path planning (Yu and Lavalle, 2015; Sharon et al., 2015; Stern et al., 2019) or navigation (Fox et al., 1997; van den Berg et al., 2011; Snape et al., 2011; van den Berg et al., 2008; Guy et al., 2010; Bouraine et al., 2014); order picking (Wurman et al., 2008; Hazard and Wurman, 2006); sustainable agricultural foraging (Song and Vaughan, 2013); and more (Kaminka et al., 2010).

Necessarily, the robots share resources (at the very least, the space of their work area), and thus, a fundamental challenge is the challenge of *multi-robot coordination*. As robots cannot act completely independent of others, they must coordinate their actions with other robots in order to avoid and resolve conflicts over resource use. Such coordination necessarily introduces some overhead into the workings of the robots, either by design or by *ad hoc* necessity.

Multi-robot coordination, therefore, both *supports* and *competes* with the achievement of the goals of the robots. Managing the coordination is a necessary component of multi-robot systems and can be done in a variety of ways. Distributed approaches that rely on joint decision-making by the robots [e.g., Gage, 1992; Parker, 1998; Kaminka and Frenkel, 2005; Xu et al., 2005; Zlot and Stentz, 2006; Vig and Adams, 2006; Kaminka and Frenkel, 2007; Kaminka et al., 2007; Dias and Stentz, 2000; Dias et al., 2004; Gerkey and Mataric, 2002; Gerkey and Matarić, 2004; Goldberg et al., 2003; Farinelli et al., 2006; Parker and Tang, 2006; Tang and Parker, 2007; Liemhetcharat and Veloso, 2013; Sung et al., 2013] require high communication availability and the capability of robots to assess not just their own state but also those of others. When such high-bandwidth communications are possible, these approaches can be very effective.

Under settings in which communications are limited in bandwidth and range (e.g., as the number of robots in a group increases), swarm robotics methods offer a promising approach to manage the coordination between robots. Here, robots necessarily coordinate *ad hoc* and *locally*, with little or no communications (Hamann, 2018; Hénard et al., 2023). Swarm robotics approaches have been applied various tasks, some similar to those discussed above: coverage (Batalin and Sukhatme, 2002; Osherovich et al., 2007); foraging (Goldberg and Matarić, 1997; Rybski et al., 1998; Balch, 1999; Vaughan et al., 2000; Zuluaga and Vaughan, 2005; Rosenfeld et al., 2008; Kaminka et al., 2010; Douchan and Kaminka, 2016; Douchan et al., 2019); and flocking*,* formation maintenance*,* and collective motion (Balch and Hybinette, 2000; Mataric, 1994; Moshtagh et al., 2009; Bastien and Romanczuk, 2020).

With few exceptions (see Section 2 for a discussion), swarm robotics research has investigated settings in which swarms are homogeneous; every robot has the same capabilities as others. Ignoring stochastic elements in perception, actuation, and decision-making components, different robots would respond in an identical manner, given the same local state in which they find themselves.

In this paper, we show how swarms can perform better when they self-adapt and specialize so that their behavioral roles become heterogeneous: given the same settings, different robots in the swarm learn to respond differently.

We focus on spatial coordination in swarm foraging. This is a canonical task for swarm robotics researchers, with many practical applications (see Section 2). We may model this task as an extensive-form fully cooperative game, in which the swarm goal is an additive function of individual contributions (collected items) (Kaminka et al., 2010). As robots cannot share the same spot at the same time and must avoid and resolve collisions, they must coordinate *spatially*, acting so as to not collide and continue their task normally if a collision occurs. Theoretically, if robots could predict future collisions and their effects, they could use such a model to make optimal collision-avoidance decisions. In practice, individual robots cannot coordinate or communicate globally and, thus, cannot select actions that are optimal for the swarm as a whole.

To compensate for missing global information, Kaminka et al. (2010) presented a multi-agent independent-learner reinforcement learning approach, where a reward function, called the effectiveness index (EI), uses only local information: the time between collisions, which is easily measured by each robot independently. Robots can individually and independently use EI to adapt their collision-avoidance strategies, dynamically diversifying their behavioral responses.

Unfortunately, although the use of EI proved effective in some cases, its effectiveness is brittle (as we show). All too often, robots learned policies that minimize their individual time spent on collisions (improving their own EI rewards) but at the expense of others. This degraded the performance of the swarm as a whole. In such cases, the individual and collective utilities are said to be *mis-aligned*.

To address this, we re-examine how swarm-wide (global) utility is related to individual actions. First, we show that the total coordination overhead of the swarm (total time spent on collision avoidance, rather than foraging *per se*) is directly related to the total utility of the swarm: the less collective overhead, the more items collected. Then, we transform the extensive-form game to a fully cooperative repeated game with an unknown horizon. Treating every collision as a stage in the repeating game, we show that this collective overhead is bounded by the *total EI of all robots* over all stages. These two results are conjectured, but unproven, in previous work.

We then derive an *aligned* individual reward function, called the *aligned effective index* (AEI) . This derivation is done from first principles: given the total EI (a global measure), we derive for each robot its marginal (individual) contribution to this value. This is done by having each robot estimate the swarm utility when it is a member of the swarm and when—hypothetically—it is not (a counterfactual). This derivation step follows difference-reward formulations (Tumer et al., 2002; Tumer et al., 2008) but differs from them in the assumptions required for estimating the counterfactuals for physical robots that only measure time. The resulting individual reward— AEI —has the property that each individual can estimate the impact of its decisions on the swarm: individual improvements translate to swarm improvements. Although AEI is derived anew, it provably generalizes EI as introduced in earlier work, adding to it a component that computes the effect of counterfactual robot absence. Different assumptions on this computation lead to bounds on AEI from above and below, which we present. Although the theoretical analysis clarifies assumptions and principled results, experiments with real and simulated robots highlight gaps with respect to the reality of robots. We explore several experimental settings (simulated and real robots), using various approximations of AEI and using both discrete-time and continuous-time Q-Learning algorithms. The experiments empirically demonstrate the efficacy of the approach in practice.

The results show that in the general case, the swarm as a whole achieves maximal results when its members become specialized through learning, i.e., they become *behaviorally diverse*: their responses to potential collisions differ, and it is that diversity that achieves maximal results. This conclusion complements those of others, investigating mechanical diversity or capability diversity in swarms (Dorigo et al., 2012; Kaminka et al., 2017; Berumen et al., 2023; Adams et al., 2023).

This paper is organized as follows: Section 2 provides background and motivation for the foraging the task, as well as a review of related work; Section 3 details the theoretical model; Section 4 discusses its approximation in the reality of robotics in practice; Section 5 presents the results from extensive simulation and real robot experiments; and Section 6 concludes with a discussion on the implications and scope of the work.

## 2 Motivation and background

We discuss the background and context for this study. First, we motivate the focus on multi-robot swarm foraging and commercial variants in Section 2.1. We then present a view of swarm foraging from the perspective of the single swarm member (Section 2.2). This allows us to place previous and existing work in context and also to present the opportunity for using learning for improving foraging. Section 2.3 focuses on investigations of this opportunity and their relation to the techniques reported here.

### 2.1 Swarm foraging: an exemplary swarm task

The motivation for our work arises from the scientific study of a canonical multi-robot task: *foraging* (Balch, 1999; Winfield, 2009; Zedadra et al., 2017; Lu et al., 2020). This is a task where a group of robots is deployed to repeatedly search for objects of interest (*items*) and, when found, for transporting them to one or more collection points (*homes*). Foraging is a canonical multi-robot problem because it raises challenges in multiple aspects of multi-robot systems:

• Management of communications between robots, e.g., with respect to where items may be found. Communications are often non-existent or limited in range and bandwidth; they may be stigmergic, as in the case of ant trail pheromones.
• Effects of population changes (robot death/birth) and various types of individual failures.
• Scalability of methods as groups grow in size
• Collision handling and avoidance as robots inevitably crowd around home locations and sometimes in areas with high item density.

We cannot do justice to a full survey of multi-robot coordination, even if we limit ourselves to foraging. Some surveys of interest on swarms in general (Hamann, 2018; Dorigo et al., 2021; Hénard et al., 2023) and foraging in particular (Winfield, 2009; Zedadra et al., 2017; Lu et al., 2020) may be found elsewhere. We discuss the most closely related work below.

Our focus is on a swarm version of foraging, where robots do not rely on communications for coordination and have little knowledge of the state of others other than their bearing within some limited local range. In other words, we assume that robots can find items, transport them, and repeat the process. They can sense the bearing (angle) to others within a limited range so that they may attempt to avoid collisions or resolve them if they occur. Other than this sensing capability, we only assume they have their own internal clocks (which are not globally synchronized), so they may, for instance, measure the time from a previous collision.

The robots have mass and cannot pass through each other, in contrast to theoretical investigations of so-called “particle agents” (Löffler et al., 2023). We make no assumption as to the self-mobility of the items themselves, although in our experiments, the items were static [see the studies by Rosenfeld et al., 2008; Hahn et al., 2020 for examples of foraging while needing to track targets].

Foraging has largely been investigated in settings where transporting an item requires a single robot, and we maintain this assumption here. However, other investigations have broken away from this assumption (and others noted above). Adhikari (2021) and Lee et al. (2022) discussed dynamic robot chains (“bucket brigades”), in which robots pass items from one to the other to avoid congestion, utilizing multiple robots even for a single item. Pitonakova et al. (2014), Ordaz-Rivas et al. (2021), and Ordaz-Rivas and Torres-Treviño (2022) addressed collective transport tasks in foraging, where multiple robots are required in order to move a single object. From this perspective, AVERT (Amanatiadis et al., 2015) is also a related system. It is a four-robot system designed to transport wheeled vehicles by having each robot attaches itself to a wheel, lifting the vehicle and carrying it together.

Almost all investigations of foraging swarms, including ours reported here, are of fully cooperative systems, where robots are assumed to be cooperative, and coordination is a challenge that arises out of their limited capabilities. This assumption stands at the basis of many applications of foraging for physically searching areas (Schloesser et al., 2021; Aljalaud and Kurdi, 2021), search-and-rescue operations (Francisco et al., 2018; Suarez and Murphy, 2011; Pham and Nguyen, 2020), and humanitarian mine clearance (McGuigan et al., 2022). Albiero et al. (2022) surveyed a few dozen investigations of agricultural applications of swarm robotics, of which a large number discuss foraging variants or highly related technologies. Dorigo et al. (2021) examined new application areas for swarms in general, foraging in particular. These include future applications in precision agriculture (e.g., harvesting), industrial monitoring and inspection, civil protection and response to natural disasters, and molecular robotics for medicinal and clinical intervention.

Recent studies break away from the assumption of fully cooperative swarms. They are motivated by future applications of foraging, where robots are self-interested (e.g., manufactured or deployed by different organizations). In such cases, the robots have to be incentivized to cooperate (Van Calck et al., 2023) and coordinate under conditions requiring privacy protection (Ferrer et al., 2021) and proof-of-work (Pacheco et al., 2022).

One specific application of foraging, *order picking*, is of particular interest here. It is a highly successful commercially significant variant of foraging, where robots collect items in a logistic warehouse, in order to fulfill customer orders (e.g., arriving via the web) (Wurman et al., 2008; Hazard and Wurman, 2006). Automated robotic order picking is one of the key technologies developed by Amazon Robotics, after it was acquired by Amazon in its takeover of Kiva Systems (for 775 million dollars; at the time, Amazon’s second-largest acquisition). This system was built to replace most human labor in a logistics warehouses^1^. In such settings, robots must engage in spatial coordination, e.g., while moving in the passageways along shelves, or when arriving at the packing stations with the collected items.

Order picking is a complex task and is interesting from a number of different technological perspectives. From a pure swarm perspective, it may be looked at as a form of foraging: robots individually look for items of interest, pick them up, and bring them to a target area. From a centralized control perspective, order picking can be viewed as a particularly challenging continual N -robot motion planning task, where a central server computes non-colliding paths for the robots.

From a scientific point of view, both perspectives raise interesting challenges worthy of investigation. Centralized algorithms for planning multi-robot paths can guarantee optimal paths free from collisions. However, such planning is computationally intractable (Yu and Lavalle, 2015; Sharon et al., 2015; Stern et al., 2019). In contrast, swarm methods are simple to deploy and robust to population changes, although typically sacrificing the ability to prevent all collisions. This raises the need for online collision-handling methods, which, in swarm settings, are often *myopic* and far from optimal. Necessarily, they respond to a collision with little or no ability to consider future collisions and inevitable crowding (e.g., around the target areas).

We observe that regardless of the scientific lens through which we examine order picking, we find that on-board, fully autonomous collision avoidance is a strictly *necessary* component. There are two reasons for this (Wurman et al., 2008):

• First, human workers may move about the warehouse—neither do they follow trajectories planned for robots (Thrun et al., 2000) nor can they be relied on to avoid collisions in a manner compatible with the robots’ choices. Moreover, human involvement may be needed in other applications as well (Schloesser et al., 2021).
• Second, even under the assumption that a planning algorithm generates perfect trajectories for the robots, and no humans are about, the possibility of electro–mechanical and communication failures (even non-catastrophic failures, such as simply slowing down as battery levels decrease) requires the robots to have on-board collision-avoidance and re-planning capabilities (Simmons et al., 1997).

In reality, therefore, robots deployed for order picking essentially carry out swarm foraging, albeit perhaps more guided in their search for items and when moving toward home. In the early version of the Kiva system, as captured by the alphabet soup simulator published by Kiva engineers, each robot was responsible for its own path-planning and collision-avoidance responses.

### 2.2 Improving foraging by improving collision avoidance

**FIGURE 1:**
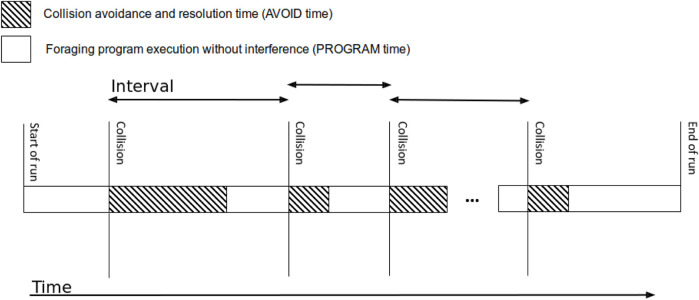
Single robot’s timeline.

Figure 1 shows a perspective [also described by Kaminka et al. (2010); Douchan et al. (2019), although using somewhat different terminology] on the execution timeline from the perspective of a single robot engaged in foraging. The robot begins by executing its foraging activity, stopping when a spatial conflict occurs (e.g., a collision is imminent). It then selects a collision-handling method, which executes for a time. When the collision is averted, the robot can switch back to carrying out its foraging until another collision is imminent. This repeats until the robot task is externally terminated (e.g., by the need to recharge). Each interval between collisions is split into two, termed the *avoidance time* (spent by the robot actively coordinating—shown in gray) and the *program time* (no need to coordinate; the robot focuses on its task).

This view of the robot’s timeline allows us to position our work with respect to others. First, many foraging methods focus on improving the productivity of the *program* phase, where the search (for items or home) takes place. This can be done by having robots (i) plan their paths better (Duncan et al., 2022; Cheraghi et al., 2020; Nuzhin et al., 2021; Jin et al., 2020), assuming some localization capabilities, or (ii) communicate information relevant to improving the search (Hoff et al., 2010; Sung et al., 2013; Pitonakova et al., 2014; Alers et al., 2014; McGuigan et al., 2022; Adams et al., 2023; Salman et al., 2024).

A second set of investigations focus on attempting to optimize an entire cycle (avoidance and program), by restricting the behavior of the robot during both program and avoidance such that collisions are minimized, and their resolution is relatively fast. For instance, Schneider-Fontan and Matarić (1998) reported on an algorithm that pre-allocates robots to different territories. Each robot operates in its territory but has the ability to pass objects to another, thus creating a bucket brigade-like structure. They also discussed re-allocating the territories once a robot fails. Goldberg and Mataric (1997) compared several different approaches to this task, measuring the amount of *interference* between the robots, as a tool for choosing an appropriate approach (see more on interference below).

A third independent direction attempt to shorten the time spent on avoidance so as to free up time for program. The most direct approach here is to improve the collision avoidance algorithm.

Not all collision-avoidance algorithms are a good fit for swarm foraging. For example, algorithms in the *reciprocal velocity obstacle* (RVO) class of navigation methods (Snape et al., 2011; Guy et al., 2010; van den Berg et al., 2008) plan ahead based on the space of admissible relative velocities to nearby obstacles and robots. They therefore assume knowledge of others’ velocities and shapes—a challenging task in many cases (e.g., when using vision only). To guarantee collision-free paths (within a specific horizon), the optimal reciprocal collision avoidance (ORCA) algorithm (van den Berg et al., 2011) also requires that all agents use ORCA, which fails when humans are involved. In contrast, the *passively safe partial motion planning* (PassPMP) algorithm (Bouraine et al., 2014) provides some guarantees on collision safety, without making such assumptions. This comes at a cost of non-trivial computation of predicted trajectories.

A related approach, presented by Danassis and Faltings (2018), is called $CA^{3}NONY$ and intended for domains where an optimal behavior will be to anti-coordinate^2^, i.e., that each agent must choose an action that differs from other agents’ actions in order for the outcome to be optimal. Here, agents are being *courteous*: If an agent collides with another agent, i.e., chooses the same resource at the same context, it backs off from this choice with a constant probability. In addition to this social convention, the agents maintain a distributed bookkeeping scheme that prevents them from monopolizing resources, causing each agent to choose only one resource for one context. Although this algorithm guarantees optimal behavior, it assumes that the reward is shared between all agents, an assumption that breaks with no communications between the agents.

Other algorithms appear to work relatively well in swarm robots, in practice. However, these offer no guarantees at all. These are essentially reactive algorithms that respond to a collision, with no or very little planning with respect to the task goal of the robot or the group, i.e., these are necessarily *myopic* algorithms. On the other hand, such algorithms are extremely simple to implement and use (both in practice and from a computational perspective) and are generally task-independent (because they do not use information about the goals of the task).

We use several such myopic algorithms in this research. The *dynamic window* algorithm (Fox et al., 1997) is a coordination method that uses limited planning in the space of admissible velocities. This method is capable of making decisions based not only on external constraints like obstacles and other robots but also on internal constraints like maximal velocity and acceleration. We use a dynamic window variant as one of the algorithms in the experiments. One reactive algorithm is the *noise* algorithm presented by Balch and Arkin (1998). Given a collision, the robot repels itself away from the collision, with some directional noise. Rosenfeld et al. (2008) described the *repel* method. As the name suggests, once a robot collides with another robot, it repels itself backward for an arbitrary time interval or distance.

More sophisticated algorithms introduce stochasticity into the decision-making. A reactive algorithm named *aggression* was described by Vaughan et al. (2000) and improved by Zuluaga and Vaughan (2005). When robots use this coordination method, the robot with the highest “aggression factor” gets the right of way, while the other backs off.

It is now understood that while each method is effective in some settings, no method is always effective (Rybski et al., 1998; Rosenfeld et al., 2008; Erusalimchik and Kaminka, 2008; Douchan and Kaminka, 2016). The results in foraging show that the swarm-wide utility—the number of collected items of a specific coordination method—depends on the density of the system. For all methods, the system-wide utility declines once some density is reached. However, the density in which this occurs differs from one method to the next. Certainly, some methods do better than others, but none are superior to others in all densities.

The performance of the swarm as the group grows in size mimics the *law of marginal returns* in economics: adding more robots does not necessarily increase productivity. Goldberg and Mataric (1997) attempted to capture the cause for this, by defining *interference*, a global signal which varies in the working space of the system denoting how much robots interfere with each other, e.g., due to lack of coordination. Later, Lerman and Galstyan (2002) drew a theoretical connection between interference and task performance. This suggests that if robots act based on the global interference signal, they might improve productivity. The problem is that in practice, this signal cannot be individually computed (as it involves internal measurements from each robot) or made public without communications.

### 2.3 Learning to coordinate in handling collisions

Inspired by the study of interference and attempting to find a way to use it despite not having access to the global (group-wide) information required, Rosenfeld et al. (2008) showed that in foraging with a fixed group size, areas of high density of robots correlate negatively with group performance. In addition, the higher the cost robots invest on coordination methods the less the group performance will be. They defined the *likelihood of collision* around a robot as the ratio between the area of a circle of fixed radius around it and the total area robots take inside this circle. They represented the cost of coordination by the *combined coordination cost* (CCC), a weighted average of all coordination costs of a robot like time and fuel. They showed a strong negative correlation between the CCC and group performance for a fixed group size.

Rosenfeld et al. (2008) then proposed an offline adaptive algorithm for the problem of multi-robot coordination, based on their CCC measure. This algorithm arbitrates between a set of coordination methods by using methods with larger CCC when the likelihood of collision is high and methods with lower CCC when the likelihood of collision is low. It does so by sorting the set of coordination methods from the one with lowest to the one with highest CCC and sets thresholds based on the likelihood of collision to determine what method to choose. The adaptation was done by tuning the aforementioned threshold. They used two separate variants for this adaptation: hill climbing and gradient learning; each one of them tunes the thresholds differently based on the group performance. The CCC measure was not developed theoretically, despite the empirical success of using it as the basis for learning (offline).

More generally, there is much work on utilizing learning to improve multi-robot (and multi-agent) coordination, mostly focusing on *multi-agent reinforcement learning*, which is often used in the context of planning and decision-making. Indeed, this is the approach we take in this paper: to improve coordination by using learning to adjust which reactive coordination method is to be used in each conflict. We only describe it here in brief and refer the reader to previous studies (Kapetanakis and Kudenko, 2002; Hernandez-Leal et al., 2019; Kober et al., 2013; Zhang et al., 2021; Kuckling, 2023; Fatima et al., 2024) for a deeper explanation. There are several investigations that are closely related to this approach, which we describe below in detail.

Claus and Boutilier (1998) showed different variations of RL techniques in multi-agent domains and the difficulties that arise when using them. They divide learners into two different types: independent learners (IL) and joint-action learners (JAL). ILs learn actions with no knowledge about the actions of other agents, while JALs learn with knowledge about the actions of all other agents. To ground RL use in multi-agent systems, Claus and Boutilier discussed learning in the context of game theory models. They showed that even simple RL algorithms lead to non-intuitive results, depending on the settings of the game. In particular, they examined both IL and JAL agents in several identical-interest matrix games (where, in every action profile, every agent gets the same utility). For both ILs and JALs, they showed that the agents converge to a Nash equilibrium, which is sub-optimal in terms of welfare. They also show that different learning parameters such as the learning rate or exploration rate can make the system converge to different equilibrium points. As we are interested in maximizing the global utility (the group goal), this is a serious challenge, which has been undertaken in many investigations.

Kaminka et al. (2010) attempted to utilize an online adaptation mechanism for the same purpose. They introduced the first version of the reward function we discuss in this paper, the EI. This basic version measured the ratio between the resources (including time) spent in collision avoidance (avoidance time, in Figure 1) and the total resources spent in a single interval between collisions (sum of the avoidance and program time in the interval). Using a stateless Q-learning variant (Claus and Boutilier, 1998) with this basic EI as a reward and using a large learning rate so as to adapt quickly to changing conditions, they demonstrated successful foraging in many different settings. Their experiments revealed that while the robots did not converge to a specific policy (individually, i.e., robots often changed their selected action), their choices are heterogeneous and often lead to improved results (e.g., compared homogeneous policies or random mixed choices).

Despite the empirical success of the EI measurement using reinforcement learning, it comes with no guarantees. Indeed, our research work began by applying the framework to the pick ordering domain, which turned out to be not at all trivial or necessarily successful (Douchan and Kaminka, 2016). We therefore sought to ground the EI in theory and, along the way, developed a more general EI reward function; the EI introduced by Kaminka et al. (2010) is strictly a special case. The general EI introduced in this paper provides guarantees up to explicit assumptions, as well as a thorough discussion of approximation methods that can be used in practice and are motivated by the theory. It adds a component by which the individual agent estimates its effect on the swarm, allowing the general EI reward to align the individual and swarm utilities.

Godoy et al. (2015) described a method using reinforcement learning techniques with ORCA (van den Berg et al., 2011). It improves on either using only ORCA or only reinforcement learning. They presented the ALAN framework that uses a reinforcement signal composed of two factors: a goal-oriented and politeness factor. The goal-oriented factor is based on the direction cosine of the velocity vector of the robot and the displacement vector of the goal from the robot. The politeness factor is based on the vector cosine between the preferred velocity vector, and the vector ORCA will output in the current robot’s situation. The final reinforcement signal for the ALAN framework is a weighted sum of the goal-oriented factor and politeness factor. This work has both similarities and dissimilarities to our work. In a similar manner to our work, this work uses reinforcement learning in order to choose the best action in any given time. However, ALAN can only choose between alternatives within ORCA and does not provide guarantees on performance, as we do here.

Wolpert and Tumer (1999) described the COIN framework, which models multi-agent systems where agents work to maximize global utility but with little or no communications between them. They show that if agents can estimate the *wonderful life utility*—how the agent’s actions (or lack thereof) impact global utility—then it is possible to use reinforcement learning to improve global utility in a guaranteed manner, in various multi-agent domains (Tumer et al., 2002; Agogino and Tumer, 2008; Wolpert et al., 1999). However, this relies on knowing the global utility and/or the value (payoff) of others’ actions. In practice, this is often not possible, so approximations are made (Tumer et al., 2008; Devlin et al., 2014). In an earlier version of the work reported here (Douchan et al., 2019), we built on the COIN work by showing how to approximate the wonderful life utility in practice, in multi-robot swarm settings. We also briefly discussed a connection to game theory. Here, we extend these results and focus on the heterogeneous nature of the resulting optimal swarms. In addition, necessarily, because we work with physical robots, and given the focus on using timing information, the approximations we take here are different from those made elsewhere; they are discussed in context in the next sections.

## 3 Swarming in (game) theory

We begin in Section 3.1 by introducing an abstract game-theoretic model of multi-robot tasks carried out by a swarm of robots. We then make incremental modifications to this abstract model, to bring it closer to the reality of physical interacting robots, when the robots cannot communicate (Sections 3.2, 3.3). Finally, in Section 3.4, we address the challenge of learning optimal actions according to the game-theoretic model we introduced. For the benefit of the reader, we include a nomenclature of the symbols in Appendix A.

### 3.1 Swarm tasks as extensive-form fully cooperative games

When considering the task multiple robots (each engaging in its own coordination method arbitration), we follow Kaminka et al. (2010) in representing the task as an extensive form game between n robots. The extensive form game represents every possible outcome as a function of the sequence of parallel coordination actions taken by all robots in every collision during the run. In this context, the outcome is the utility of each of the robots in the allotted time.

The root node of the game tree represents the first collision. Given that there are n robots, the first n layers of the game tree will each represent a robot and its possible actions in the first collision. This is because we focus on non-communicating coordination methods, and thus, we treat each collision as having no information about the actions and utilities selected and gained by other robots.

The actions independently taken by players are coordination methods. The gains (payoffs) from taking them and the costs that they entail differ between robots and between collisions but are theoretically accounted for. Each action takes time.

The next m layers represent the second collision in the same manner, and the pattern repeats until a terminal node is reached—when the time for the task is done. A terminal node represents the end of the game (task) and holds the sum of the utility of each player. Since different actions can yield different time intervals between collisions, terminal nodes can each be of different depths depending on the sequence of collisions (and associated joint actions chosen) during the game. Each such sequence is represented as a path in the game tree.

Each terminal node will hold a vector of numerical values representing the utilities of each robot in the system. As this is a cooperative task, we are interested in the sum of these utilities—in foraging this would translate to the number of items collected by all swarm members, together.

**FIGURE 2:**
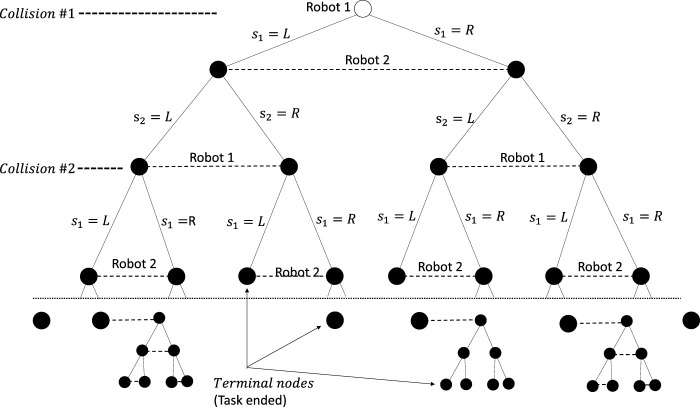
Two-player two-action task run represented as an extensive-form game for the action sets $S_{1}=S_{2}=\left\{L,R\right\}$ . Not all terminal nodes have the same depth as different joint actions taken by the players can lead to more or less collisions.

A two-player two-action example of such an extensive-form game is shown in Figure 2. It shows several paths from the root node to the terminal nodes.

### 3.2 From extensive-form game to normal-form games

The extensive-form model of a task run represents every possible outcome of the task run. This is only of theoretical value as no robot—or their designers—can predict the outcome of future collisions, or their timing, or their impact on global payoffs. In reality, robots only know their history of previous collisions, and the immediately imminent collision. Indeed, in swarm settings, robots cannot know of the other robots’ choices (which theoretically affects their own), and thus, even this information is hidden from them.

In order for robots to make decisions based only the history and current collision, we must draw a connection between the global final utility (payoff) theoretically reached using the extensive-form game and the sequence of collisions in which the robots make collision-resolution choices. Robots may then rely on signals that are obtained during a joint collision.

To do this, we take an intermediate step and show how the extensive-form game can be expressed as a sequence of normal-form games, each representing a single joint collision. We define the following symbols (see also nomenclature in Table A1):

• $s_{i}^{j}$ : robot i ’s action at the j th collision. $s^{j}$ : joint action at collision j .
• $h_{i}^{j}=\left(s_{i}^{1},s_{i}^{2},\ldots ,s_{i}^{j}\right)$ : robot i ’s history of actions until the j th collision inclusive. History of all robots’ actions until j inclusive: $h^{j}$ .
• The *cost* incurred by robot i at the j th collision is denoted as $c_{i}^{j}$ .
• The *gain* by robot i at the j th collision is denoted as $g_{i}^{j}$ .
• $u_{i}^{j}=g_{i}^{j}-c_{i}^{j}$ : the *utility* of robot i at the j th collision.
• U : the *global utility* of the entire robot swarm during the whole run.
• C : the number of collisions during the whole run.

We start with the most general case where outcomes of a robot at the j th collision may depend on the entire history of play of all the robots up until collision j inclusive. This means that $u_{i}^{j},g_{i}^{j},c_{i}^{j}$ are all functions of $h^{j}$ . U now depends on the entire history of play. Given that the number of collisions for the whole task run is C , U will be a function of $h^{C}$ and will be defined as the sum of utilities of every robot and every collision during the task run (Equations 1, 2).

$$U\left(h^{C}\right)\;:=\underset{i\in N}{\sum }\overset{C}{\underset{j=1}{\sum }}u_{i}\left(h^{j}\right)=\underset{i\in N}{\sum }\overset{C}{\underset{j=1}{\sum }}\left(g_{i}\left(h^{j}\right)-c_{i}\left(h^{j}\right)\right).$$ (1)

We can look at each joint collision as a normal-form (matrix) game representing the outcomes of this collision only, rather than the whole task run. For the j th collision, the player set of this matrix is the set of robots performing the task, and the action set of each robot is its set of available coordination methods for this collision. Given the history of joint actions played up until collision j (inclusive), $h^{j}$ , the payoffs of this matrix will be the sum of the utilities of the robots obtained only for the j th collision $\sum_{i\in N}u_{i}\left(h^{j}\right)$ as a function of the history of play. We call this matrix the *folded game matrix*.

**FIGURE 3:**
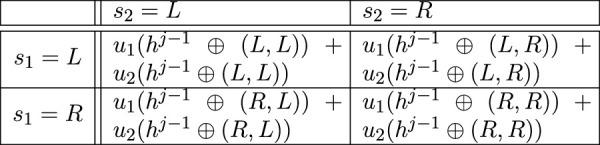
Example of a two-player two-action folded game matrix for the action set $S_{1}=S_{2}=\left\{L,R\right\}$ .

We define the ⊕ operator between a play history and a new joint action to be the concatenation of the new joint action to the history. For $h^{j-1}=\left(s^{1},\ldots ,s^{j-1}\right)$ and $s^{j}$ , $h^{j}=h^{j-1}⊕s^{j}=\left(s^{1},\ldots ,s^{j-1},s^{j}\right)$ . Figure 3 provides an illustration.

### 3.3 Global utility and folded matrices

Robots in a system have limited sensing and communication capabilities. They are unable to know the utilities of other robots, even in the same joint action. Indeed, each robot does not even know how its own action affects its own immediate utility. The only information available to a robot is from its own sensors and internal state memory.

In particular, the robot i can measure time. It can measure the time—denoted $A_{i}\left(s\right)$ —it has spent in collision avoidance after having executed a collision avoidance procedure s . It can also measure the time—denoted $P_{i}\left(s\right)$ —spent making progress on its task, undisturbed by others, once the collision is resolved.

We formally tie the avoidance and program times of the collision to the utility of the robot resulting from the collision. To do this, we assume that individual gains in avoidance time are zero (since a robot in avoidance time is handling a collision), and therefore, gains occur only in program time: $g_{i}\left(h^{j}\right)=g_{i}\left(P_{i}\left(h^{j}\right)\right)$ . We will further assume that the gains are proportional to the program time, given the history of play $h^{j}$ : $g_{i}\left(h^{j}\right)=\alpha P_{i}\left(h^{j}\right)$ , where α is a positive constant. We also assume that costs are constant, $c_{i}\left(h^{j}\right)=\beta \left(A_{i}\left(h^{j}\right)+P_{i}\left(h^{j}\right)\right)$ , where β is a positive constant. Therefore, by substituting g,c by their interval proxies using α,β , the following holds:

By definition, Equation 1

$$U\left(h^{C}\right)=\underset{i\in N}{\sum }\overset{C}{\underset{j=1}{\sum }}\left(g_{i}\left(h^{j}\right)-c_{i}\left(h^{j}\right)\right),$$

$$=\underset{i\in N}{\sum }\overset{C}{\underset{j=1}{\sum }}\left[\alpha P_{i}\left(h^{j}\right)-\beta \left(A_{i}\left(h^{j}\right)+P_{i}\left(h^{j}\right)\right)\right].$$ (2)

#### 3.3.1 Global utility and coordination overhead

Rosenfeld et al. (2008) empirically demonstrated that there is a strong negative correlation between coordination costs (the avoidance time in our case) and swarm performance. The more a robot, or a group of robots, spends time carrying out the task (program time) and less on coordination (avoidance time), the higher is and the higher their performance. Equation 2 formally shows this relationship.

We distinguish productive intervals (P) from coordination (collision avoidance) intervals (A) in Equation 2. Given a task run $h^{C}$ (a sequence of C joint actions by the swarm members), we define the coordination overhead of the swarm is defined as follows (Equation 3):

Definition 1 The *coordination overhead (CO)* is the total amount of time the system was in avoidance time divided by the total time invested in the task run:

$$CO\left(h^{C}\right)≔\frac{1}{T}\underset{i\in N}{\sum }\overset{C}{\underset{j=1}{\sum }}A_{i}\left(h^{j}\right).$$ (3)

We show that U is a linear decreasing function of CO , i.e., minimizing CO is maximizing U . In the following, n=|N| , i.e., it is the number of robots.

Theorem 1 Given the assumptions on the cost and gain, U is a linear decreasing function of CO .

Proof.

$$\begin{array}{ll} U\left(h^{C}\right) & =\underset{i\in N}{\sum }\overset{C}{\underset{j=1}{\sum }}\left[u_{i}\left(h^{j}\right)\right]=\underset{i\in N}{\sum }\overset{C}{\underset{j=1}{\sum }}\left[g_{i}\left(h^{j}\right)-c_{i}\left(h^{j}\right)\right]\\ & =\underset{i\in N}{\sum }\overset{C}{\underset{j=1}{\sum }}\left[\alpha P_{i}\left(h^{j}\right)-\beta \left(A_{i}\left(h^{j}\right)+P_{i}\left(h^{j}\right)\right)\right]\\ & =\underset{i\in N}{\sum }\overset{C}{\underset{j=1}{\sum }}\alpha P_{i}\left(h^{j}\right)-\underset{i\in N}{\sum }\overset{C}{\underset{j=1}{\sum }}\beta \left[A_{i}\left(h^{j}\right)+P_{i}\left(h^{j}\right)\right]\\ & =\alpha \underset{i\in N}{\sum }\overset{C}{\underset{j=1}{\sum }}P_{i}\left(h^{j}\right)-\beta \underset{i\in N}{\sum }\overset{C}{\underset{j=1}{\sum }}\left[A_{i}\left(h^{j}\right)+P_{i}\left(h^{j}\right)\right]. \end{array}$$

Since T is the sum of all cycle lengths of any of the ${robots}^{\prime }$ task run, we can write $T=\sum_{j=1}^{C}\left(A_{i}\left(h^{j}\right)+P_{i}\left(h^{j}\right)\right)$ for any robot i . Thus,

$$\begin{array}{ll} & =\alpha \underset{i\in N}{\sum }\overset{C}{\underset{j=1}{\sum }}P_{i}\left(h^{j}\right)-\beta \underset{i\in N}{\sum }T\;\left(T\;\text{total time, identical for all robots}\right)\\ & =\alpha T\frac{\underset{i\in N}{\sum }\overset{C}{\underset{j=1}{\sum }}P_{i}\left(h^{j}\right)}{T}-\beta nT\;\left(n=|N|\;\text{is the number of agents}\right)\\ & =\alpha T\left(1-CO\left(h^{C}\right)\right)-\beta nT=\alpha T-\alpha TCO\left(h^{C}\right)-\beta nT\\ & =-\alpha T\cdot CO\left(h^{C}\right)+T\left(\alpha -n\beta \right). \end{array}$$

As α,β,T are positive constants in this setting, it follows that U is a linear decreasing function of $CO\left(h^{C}\right)$ .

This completes the proof. □

As a result of Theorem 1, now it is possible to look at our problem as minimizing CO rather than maximizing U . However, this does not give information about how robots should choose their actions in a way that CO is minimized.

#### 3.3.2 Coordination overhead and the folded matrices

We turn to utilizing the folded matrices as a step toward making it possible for robots to maximize U (by minimizing the swarm’s CO ). To do this, we re-examine the sequence of normal-form games making up the history of agent decisions.

We follow Kaminka et al. (2010); Douchan and Kaminka (2016) in making a Markovian assumption that for every collision, the outcomes of the robots’ method selection depend only on the current joint action performed and not on the history of all joint actions performed. This means that the outcome of any collision, given a collision-avoidance action s , depends only on the action and not the history of previous collisions. Under this assumption, variables $h^{j}\in S^{j}$ that depend on the history of joint actions played until collision j , depend only on the joint action that was played in time j , $s^{j}\in S$ . We therefore denote the avoidance time as $A_{i}\left(s^{j}\right)$ . The same applies for $P_{i},g_{i},c_{i},u_{i}$ and U .

One consequence of this assumption is that instead of the task run being a sequence of different folded-game matrices depending on the history of play, it is now a single game matrix, which is the same for every collision in the task run. In game theory, such a sequence is termed *repeating games*. As the number of games is not known in advance, these settings are formally known as *infinite-horizon* repeating games.

Minimizing CO maximizes the global utility. Since T is the sum of all cycle length of any of the robots’ task run, we can write $T=\sum_{j=1}^{C}\left(A_{i}\left(h^{j}\right)+P_{i}\left(h^{j}\right)\right)$ for any robot i . Therefore, CO can also be written as

$$CO\left(h^{C}\right)=\underset{i\in N}{\sum }\frac{\overset{C}{\underset{j=1}{\sum }}A_{i}\left(s^{j}\right)}{\overset{C}{\underset{j=1}{\sum }}\left[A_{i}\left(s^{j}\right)+P_{i}\left(s^{j}\right)\right]}.$$ (4)

Given the above, it makes sense for swarm agents to attempt to *individually* increase their own $P_{i}\left(\right)$ and minimize their own $A_{i}\left(\right)$ by selecting appropriate individual actions. Indeed, Kaminka et al. (2010)—*predating the introduction of the coordination overhead*—proposed using the ratio of avoidance time to total cycle duration (since the last conflict) as a substitute for the robot’s estimate of the swarm’s utility from taking an action s . They refer to this ratio as the EI:

$$EI\left(i,s\right)≔\frac{A_{i}\left(s\right)}{A_{i}\left(s\right)+P_{i}\left(s\right)}.$$ (5)

Kaminka et al. (2010) conjectured that individually minimizing EI is equivalent to maximizing the robot’s utility and, thus, the swarm’s utility. However, *this conjectured connection is generally incorrect*: maximizing the individual robot’s EI may mean selecting an action that increases the costs to others, resulting in overall degraded performance for the swarm. To intuitively see why this happens, imagine some foraging robots are attempting to enter the collection area to drop foraged items, while others are attempting to leave, to search for new items. Those attempting to enter should ideally back off, allowing those inside the nest to go out. However, backing off adds to the duration of the avoidance mode and reduces from the duration of the program mode. Thus, those robots are motivated to push forward. This hinders the swarm from collecting items.

Despite its lacking, the structural similarity between the individual EI (Equation 5) and the coordination overhead in its rewritten form (Equation 4) has led us to define a related measure, $EI_{\mathrm{tot}}\left(s\right)$ , the total sum of the effectiveness indices of all robots:

$$\begin{array}{ll} EI_{\mathrm{tot}}\left(s\right) & ≔\underset{i\in N}{\sum }EI\left(i,s\right)\\ & =\underset{i\in N}{\sum }\frac{A_{i}\left(s\right)}{A_{i}\left(s\right)+P_{i}\left(s\right)}. \end{array}$$ (6)

We draw a connection between $EI_{\mathrm{tot}}$ (Equation 6) and CO (Equations 3, 4). Let s* be the joint action that minimizes $EI_{\mathrm{tot}}$ :

$$s^{*}≔\underset{s}{\mathrm{arg min}}\left(EI_{tot}\left(s\right)\right).$$

Let the swarm play the joint action s* repeatedly, generating the history $h^{*}=\left(s*,s*,\ldots ,s*\right)$ . Then, $CO\left(h^{*}\right)$ will be equal to $EI_{\mathrm{tot}}\left(s*\right)$ : $CO\left(h^{*}\right)=\sum_{i\in N}\frac{C\cdot A_{i}\left(s^{*}\right)}{C\cdot \left[A_{i}\left(s^{*}\right)+P_{i}\left(s^{*}\right)\right]}=\sum_{i\in N}\frac{A_{i}\left(s^{*}\right)}{A_{i}\left(s^{*}\right)+P_{i}\left(s^{*}\right)}=EI_{\mathrm{tot}}\left(s*\right)$ .

Building on this, we show that for every sequence of joint actions, CO will be greater or equal to $EI_{\mathrm{tot}}\left(s*\right)$ . This means that in order to minimize CO , the system always needs to select s* as the joint action.

Theorem 2 For any number of collisions C and histories of play $h^{C}$ , $CO\left(h^{C}\right)\geq EI_{\mathrm{tot}}\left(s*\right)$ .

Proof.

$$\begin{array}{ll} CO\left(h^{C}\right) & =\underset{i\in N}{\sum }\frac{\overset{C}{\underset{j=1}{\sum }}A_{i}\left(s^{j}\right)}{\overset{C}{\underset{j=1}{\sum }}\left(A_{i}\left(s^{j}\right)+P_{i}\left(s^{j}\right)\right)}\\ & =\underset{i\in N}{\sum }\frac{\overset{C}{\underset{j=1}{\sum }}A_{i}\left(s^{j}\right)}{T}\\ & =\frac{1}{T}\underset{i\in N}{\sum }\overset{C}{\underset{j=1}{\sum }}A_{i}\left(s^{j}\right). \end{array}$$

We re-order the summations:

$$\begin{array}{ll} & =\frac{1}{T}\overset{C}{\underset{j=1}{\sum }}\underset{i\in N}{\sum }A_{i}\left(s^{j}\right). \end{array}$$

Defining $l_{i}\left(s\right)$ as the cycle length of robot i , given joint action s : $l_{i}\left(s\right)=A_{i}\left(s\right)+P_{i}\left(s\right)$ , we rewrite

$$\begin{array}{ll} & =\frac{1}{T}\overset{C}{\underset{j=1}{\sum }}\left[l\left(s^{j}\right)\underset{i\in N}{\sum }\frac{A_{i}\left(s^{j}\right)}{l\left(s^{j}\right)}\right]\\ & =\frac{1}{T}\overset{C}{\underset{j=1}{\sum }}\left[l\left(s^{j}\right)EI_{\mathrm{tot}}\left(s^{j}\right)\right]. \end{array}$$

Replacing $s^{j}$ with the optimal joint action s* , necessarily:

$$\begin{array}{ll} & \geq \frac{1}{T}\overset{C}{\underset{j=1}{\sum }}\left[l\left(s^{j}\right)EI_{\mathrm{tot}}\left(s^{*}\right)\right]\\ & =EI_{\mathrm{tot}}\left(s^{*}\right)\frac{1}{T}\overset{C}{\underset{j=1}{\sum }}l\left(s^{j}\right)\\ & =EI_{\mathrm{tot}}\left(s^{*}\right)\frac{1}{T}T\\ & =EI_{\mathrm{tot}}\left(s^{*}\right). \end{array}$$

This completes the proof. □

The step taken in Theorem 1 allows robots, in theory, to use measurements of time instead of global count of items picked (which in a swarm, they cannot possibly achieve). The step taken in Theorem 2 shows that under some assumption, the sequence of collisions can be treated as a repeating game with an infinite-horizon, where each stage is an identical normal-form game. Thus, determining s* , the optimal joint action in a collision, leads to optimal results for the swarm.

However, robots cannot know s* as it requires knowledge of the actions of other robots. We need to find a way to make the robots converge to s* by using internal measurements only, without requiring knowledge of coordination methods selected and utilities obtained, by other robots.

### 3.4 Optimal joint actions

We approach the challenge by finding a potential function that turns the normal-form game into a *potential game* [Monderer and Shapley (1996)]. A potential game is a normal-form game, where, for every player i , the difference in the payoff of every unilateral deviation of player i ’s action $s_{i}$ is related to the difference of a single potential function ψ(s) mapping each joint action to a scalar. ψ(s) can be seen as a global signal (not necessarily visible to the players) which depends on the joint action.

Potential games hold several important characteristics: First, they always have at least one pure-strategy Nash equilibrium. Furthermore, when players use pure strategies, an improvement in one player’s individual payoff due to changing its individual action will necessarily improve the potential function, i.e., the individual payoff and potential function are *aligned*. When players choose to maximize their individual payoffs, the system will converge to a pure-strategy Nash equilibrium, which would be at least a local optimum of the potential function.

If the robots play a potential game with potential function $EI_{\mathrm{tot}}$ , the swarm will converge to an optimal joint action in terms of $EI_{\mathrm{tot}}$ . However, $EI_{\mathrm{tot}}$ is a global function: it is not accessible to the robots. We therefore seek a reward function for each robot that is both accessible to each robot and aligned with $EI_{\mathrm{tot}}$ .

To derive a local aligned reward from $EI_{\mathrm{tot}}$ , we use the formulation of *Wonderful Life Utility (WLU)* (Wolpert and Tumer, 1999; Tumer et al., 2002), later renamed the *difference reward* (Agogino and Tumer, 2008; Tumer et al., 2008; Devlin et al., 2014). Given a global function F , the difference reward for robot i using joint action s is the difference between the F value resulting from the action with i participating and the counterfactual F value when robot i is hypothetically absent. We denote the absence of robot i as the robot choosing a *null* action denoted by $\phi_{i}$ . We denote by $s_{-i}$ the joint action of all robots excluding i . Then, $F\left(s_{i},s_{-i}\right)$ is the value resulting from the complete joint action (including all robots’ actions), and $F\left(\phi_{i},s_{-i}\right)$ is the value of the swarm when robot i is absent. Then, the difference reward of F is given by $\Delta_{i}^{F}≔F\left(s_{i},s_{-i}\right)-F\left(\phi_{i},s_{-i}\right)$ . For reinforcement learning, it is a reward that is both accessible and aligned, and highly effective (Tumer et al., 2008; Arslan et al., 2007; Marden and Wierman, 2009).

Using $EI_{\mathrm{tot}}$ as the global potential function, we define the *Aligned Effectiveness Index* (AEI) as the difference reward of $EI_{\mathrm{tot}}$ , i.e., $\Delta_{i}^{EI_{\mathrm{tot}}}\left(s\right)$ :

Definition 2 Given a joint action $s=\left(s_{i},s_{-i}\right)$ , the AEI reward of robot i is defined by

$$AEI\left(i,s\right)≔EI_{\mathrm{tot}}\left(s_{i},s_{-i}\right)-EI_{\mathrm{tot}}\left(\phi_{i},s_{-i}\right).$$

AEI is a measurement of robot i ’s marginal contribution to $EI_{\mathrm{tot}}$ , given the action s . If the robots individually select actions that optimize it, they will converge to a joint action that will, at least, be a local minimum of $EI_{\mathrm{tot}}$ due to the properties of potential games (Tumer et al., 2008; Marden and Wierman, 2009).

We derive a bounded closed-form expression of ${AEI}_{i}\left(s\right)$ :

$$\begin{array}{ll} AEI\left(i,s\right) & =EI_{\mathrm{tot}}\left(s_{i},s_{-i}\right)-EI_{\mathrm{tot}}\left(\phi_{i},s_{-i}\right),\;\;\;\left(\text{From Def. }2\right)\\ & =EI_{\mathrm{tot}}\left(s\right)-EI_{\mathrm{tot}}\left(\phi_{i},s_{-i}\right),\;\;\;\left(\text{Notation:}s=\left(s_{i},s_{-i}\right)\right)\\ & =\underset{j\in N}{\sum }\frac{A_{j}\left(s\right)}{A_{j}\left(s\right)+P_{j}\left(s\right)}-\underset{j\in N}{\sum }\frac{A_{j}\left(\phi_{i},s_{-i}\right)}{A_{j}\left(\phi_{i},s_{-i}\right)+P_{j}\left(\phi_{i},s_{-i}\right)}\left(\text{From Eq. }6\right)\\ & =\frac{A_{i}\left(s\right)}{A_{i}\left(s\right)+P_{i}\left(s\right)}+\underset{j\in N\backslash \left\{i\right\}}{\sum }\frac{A_{j}\left(s\right)}{A_{j}\left(s\right)+P_{j}\left(s\right)}-\underset{j\in N}{\sum }\frac{A_{j}\left(\phi_{i},s_{-i}\right)}{A_{j}\left(\phi_{i},s_{-i}\right)+P_{j}\left(\phi_{i},s_{-i}\right)}. \end{array}$$

We observe that one of the components here is actually EI Kaminka et al. (2010) (see Equation 6).

$$\begin{array}{ll} & =EI\left(i,s\right)+\underset{j\in N\backslash \left\{i\right\}}{\sum }\frac{A_{j}\left(s\right)}{A_{j}\left(s\right)+P_{j}\left(s\right)}-\underset{j\in N}{\sum }\frac{A_{j}\left(\phi_{i},s_{-i}\right)}{A_{j}\left(\phi_{i},s_{-i}\right)+P_{j}\left(\phi_{i},s_{-i}\right)}\;\left(\text{From Eq. }5\right). \end{array}$$

Once again, let $l_{i}\left(s\right)$ be the cycle length of robot i given a joint action s : $l_{i}\left(s\right)=A_{i}\left(s\right)+P_{i}\left(s\right)$ :

$$\begin{array}{lll} & =EI\left(i,s\right)+\underset{j\in N\backslash \left\{i\right\}}{\sum }\frac{A_{j}\left(s\right)}{l_{j}\left(s\right)}-\underset{j\in N}{\sum }\frac{A_{j}\left(\phi_{i},s_{-i}\right)}{l_{j}\left(\phi_{i},s_{-i}\right)} & \\ & =EI\left(i,s\right)+\underset{j\in N\backslash \left\{i\right\}}{\sum }\frac{A_{j}\left(s\right)}{l_{j}\left(s\right)}-\underset{j\in N\backslash \left\{i\right\}}{\sum }\frac{A_{j}\left(\phi_{i},s_{-i}\right)}{l_{j}\left(\phi_{i},s_{-i}\right)}-\frac{A_{i}\left(\phi_{i},s_{-i}\right)}{l_{i}\left(\phi_{i},s_{-i}\right)}. & \end{array}$$

Multiplying the first sum by $\frac{l_{i}\left(s\right)}{l_{i}\left(s\right)}=1$ and the second sum by $\frac{l_{i}\left(\phi_{i},s_{-i}\right)}{l_{i}\left(\phi_{i},s_{-i}\right)}$ yields

$$\begin{array}{ll} & =EI\left(i,s\right)+\underset{j\in N\backslash \left\{i\right\}}{\sum }\frac{A_{j}\left(s\right)\frac{l_{i}\left(s\right)}{l_{j}\left(s\right)}}{l_{i}\left(s\right)}-\underset{j\in N\backslash \left\{i\right\}}{\sum }\frac{A_{j}\left(\phi_{i},s_{-i}\right)\frac{l_{i}\left(\phi_{i},s_{-i}\right)}{l_{j}\left(\phi_{i},s_{-i}\right)}}{l_{i}\left(\phi_{i},s_{-i}\right)}-\frac{A_{i}\left(\phi_{i},s_{-i}\right)}{l_{i}\left(\phi_{i},s_{-i}\right)}. \end{array}$$

We remind the reader that we had assumed earlier that collisions are synchronous and involve all agents. This means that the cycle length depends only on the joint action selected and not on any specific robot. Therefore, for all pairs of robots i,j∈N and all joint actions s , $\frac{l_{i}\left(s\right)}{l_{j}\left(s\right)}=\frac{l_{i}\left(\phi_{i},s_{-i}\right)}{l_{j}\left(\phi_{i},s_{-i}\right)}=1$ . This yields

$$AEI\left(i,s\right)=EI\left(i,s\right)+\underset{j\in N\backslash \left\{i\right\}}{\sum }\frac{A_{j}\left(s\right)}{l_{i}\left(s\right)}-\underset{j\in N\backslash \left\{i\right\}}{\sum }\frac{A_{j}\left(\phi_{i},s_{-i}\right)}{l_{i}\left(\phi_{i},s_{-i}\right)}-\frac{A_{i}\left(\phi_{i},s_{-i}\right)}{l_{i}\left(\phi_{i},s_{-i}\right)}.$$ (7)

We note that all the denominators $l_{i}\left(\right)$ are durations measured or estimated (as counterfactuals) by robot i . However, to compute its reward, robot i must seemingly require knowledge of the duration $A_{j}\left(s\right)$ , which it does not know. We, therefore, seek a simplification of the above. This is done in two steps. First, we bound the value of AEI from below and above (Theorem 3 below). Then, we argue that as the number of robots increases, the bounds become tight, and thus, a simpler formula emerges.

Theorem 3
$$EI\left(i,s\right)+\frac{A_{i}^{\phi }\left(s\right)}{A_{i}\left(\phi_{i},s_{-i}\right)+P_{i}\left(\phi_{i},s_{-i}\right)}\geq AEI\left(i,s\right)\geq EI\left(i,s\right)+\frac{A_{i}^{\phi }\left(s\right)}{A_{i}\left(s\right)+P_{i}\left(s\right)}-1,$$

where $A_{i}^{\phi }\left(s\right)=\sum_{j\in N\backslash \left\{i\right\}}\left(A_{j}\left(s_{i},s_{-i}\right)-A_{j}\left(\phi_{i},s_{-i}\right)\right)$ .

Proof. Note that terms using ϕ are *counterfactuals*: they are hypothetical values, modeling the effects of robot i on others, when it is hypothetically logically absent from the collision, and unable to contribute. Two potential ways to model this assumption are (i) either that robot i was removed from the set N for the collision (Case 1 below), or that it is present but remained in collision avoidance during the entire cycle length and thus did not contribute (Case 2). In both cases, we begin with the closed form formulation for AEI(*i*,*s*), as found in Equation 7.

Case 1 Robot i hypothetically removed from N in the collision.

$$\frac{A_{i}\left(\phi_{i},s_{-i}\right)}{l_{i}\left(\phi_{i},s_{-i}\right)}=0,$$

and we may also assume that its absence shortens the swarm’s avoidance period (the collision resolution was shorter), and thus,

$$l_{i}\left(\phi_{i},s_{-i}\right)\leq l_{i}\left(s\right).$$

Therefore, continuing from step 7 above,

$$\begin{array}{ll} AEI\left(i,s\right) & =EI\left(i,s\right)+\underset{j\in N\backslash \left\{i\right\}}{\sum }\frac{A_{j}\left(s\right)}{l_{i}\left(s\right)}-\underset{j\in N\backslash \left\{i\right\}}{\sum }\frac{A_{j}\left(\phi_{i},s_{-i}\right)}{l_{i}\left(\phi_{i},s_{-i}\right)}-\frac{A_{i}\left(\phi_{i},s_{-i}\right)}{l_{i}\left(\phi_{i},s_{-i}\right)}\\ & =EI\left(i,s\right)+\underset{j\in N\backslash \left\{i\right\}}{\sum }\frac{A_{j}\left(s\right)}{l_{i}\left(s\right)}-\underset{j\in N\backslash \left\{i\right\}}{\sum }\frac{A_{j}\left(\phi_{i},s_{-i}\right)}{l_{i}\left(\phi_{i},s_{-i}\right)}\\ & \leq EI\left(i,s\right)+\underset{j\in N\backslash \left\{i\right\}}{\sum }\frac{A_{j}\left(s\right)}{l_{i}\left(\phi_{i},s_{-i}\right)}-\underset{j\in N\backslash \left\{i\right\}}{\sum }\frac{A_{j}\left(\phi_{i},s_{-i}\right)}{l_{i}\left(\phi_{i},s_{-i}\right)}. \end{array}$$

Adding using the common denominator and using $A_{i}^{\phi }\left(s\right)$ to denote $\sum_{j\in N\backslash \left\{i\right\}}\left[A_{j}\left(s\right)-A_{j}\left(\phi_{i},s_{-i}\right)\right]$ ,

$$\begin{array}{ll} & =EI\left(i,s\right)+\frac{A_{i}^{\phi }\left(s\right)}{l_{i}\left(\phi_{i},s_{-i}\right)}\\ & =EI\left(i,s\right)+\frac{A_{i}^{\phi }\left(s\right)}{A_{i}\left(\phi_{i},s_{-i}\right)+P_{i}\left(\phi_{i},s_{-i}\right)}\left(\text{Transforming back from}l_{i}\left(\phi_{i},s_{-i}\right)\right). \end{array}$$

This yields the left-hand inequality of the theorem. $A_{i}^{\phi }$ is the counterfactual change in the total avoidance time of the swarm when robot i is hypothetically not involved.

$$EI\left(i,s\right)+\frac{A_{i}^{\phi }\left(s\right)}{A_{i}\left(\phi_{i},s_{-i}\right)+P_{i}\left(\phi_{i},s_{-i}\right)}\geq AEI\left(i,s\right).$$ (8)

Case 2 Robot i ’s absence—inability to contribute—is modeled as being in collision avoidance for the entire duration of the cycle. In this case, $A_{i}\left(\phi_{i},s_{-i}\right)=l_{i}\left(\phi_{i},s_{-i}\right)$ , and therefore,

$$\frac{A_{i}\left(\phi_{i},s_{-i}\right)}{l_{i}\left(\phi_{i},s_{-i}\right)}=1.$$

As the agent is still hypothetically present, the counterfactual cycle length does not change:

$$l_{i}\left(\phi_{i},s_{-i}\right)=l_{i}\left(s\right).$$

Then, continuing from Equation 7 yields

$$\begin{array}{ll} AEI\left(i,s\right) & =EI\left(i,s\right)+\underset{j\in N\backslash \left\{i\right\}}{\sum }\frac{A_{j}\left(s\right)}{l_{i}\left(s\right)}-\underset{j\in N\backslash \left\{i\right\}}{\sum }\frac{A_{j}\left(\phi_{i},s_{-i}\right)}{l_{i}\left(\phi_{i},s_{-i}\right)}-\frac{A_{i}\left(\phi_{i},s_{-i}\right)}{l_{i}\left(\phi_{i},s_{-i}\right)}\\ & \geq EI\left(i,s\right)+\underset{j\in N\backslash \left\{i\right\}}{\sum }\frac{A_{j}\left(s\right)}{l_{i}\left(s\right)}-\underset{j\in N\backslash \left\{i\right\}}{\sum }\frac{A_{j}\left(\phi_{i},s_{-i}\right)}{l_{i}\left(s\right)}-1\\ & =EI\left(i,s\right)+\underset{j\in N\backslash \left\{i\right\}}{\sum }\frac{A_{j}\left(s\right)}{l_{i}\left(s\right)}-\underset{j\in N\backslash \left\{i\right\}}{\sum }\frac{A_{j}\left(\phi_{i},s_{-i}\right)}{l_{i}\left(s\right)}-1\\ & =EI\left(i,s\right)+\frac{\underset{j\in N\backslash \left\{i\right\}}{\sum }\left[A_{j}\left(s\right)-A_{j}\left(\phi_{i},s_{-i}\right)\right]}{l_{i}\left(s\right)}-1\\ & =EI\left(i,s\right)+\frac{A_{i}^{\phi }\left(s\right)}{l_{i}\left(s\right)}-1\;\;\;A_{i}^{\phi }\left(s\right)\text{as above.}\\ & =EI\left(i,s\right)+\frac{A_{i}^{\phi }\left(s\right)}{A_{i}\left(s\right)+P_{i}\left(s\right)}-1\left(\text{Transforming back from}l_{i}\left(s\right)\right). \end{array}$$

This yields the right-hand inequality of the theorem. Putting it together with the left-hand inequality (Equation 8 above) yields

$$EI\left(i,s\right)+\frac{A_{i}^{\phi }\left(s\right)}{A_{i}\left(\phi_{i},s_{-i}\right)+P_{i}\left(\phi_{i},s_{-i}\right)}\geq AEI\left(i,s\right)\geq EI\left(i,s\right)+\frac{A_{i}^{\phi }\left(s\right)}{A_{i}\left(s\right)+P_{i}\left(s\right)}-1,$$

thus completing the proof. □

We make the following observation with respect to the above derivation of bounds on AEI(i,s) in Theorem 3. As the swarm tends to grow in size (|N|) , the difference between the two bounds will tend towards 1 as the counterfactual removal of robot i from the collision will not affect the cycle length, under the assumption of synchronous collisions.

Formally, we conjecture that

$$\underset{|N|\to \infty }{\lim}\left[A_{i}\left(\phi_{i},s_{-i}\right)+P_{i}\left(\phi_{i},s_{-i}\right)\right]-\left[A_{i}\left(s\right)+P_{i}\left(s\right)\right]=0.$$

Proving this conjecture depends on a formal model of the counterfactual removal of a robot from a swarm collision, which is outside the scope of this paper. Lacking such a model, we use $\left[A_{i}\left(s\right)+P_{i}\left(s\right)\right]$ as the cycle length for $\left[A_{i}\left(\phi_{i},s_{-i}\right)+P_{i}\left(\phi_{i},s_{-i}\right)\right]$ . This implies

$$EI\left(i,s\right)+\frac{A_{i}^{\phi }\left(s\right)}{A_{i}\left(s\right)+P_{i}\left(s\right)}\geq AEI\left(i,s\right)\geq EI\left(i,s\right)+\frac{A_{i}^{\phi }\left(s\right)}{A_{i}\left(s\right)+P_{i}\left(s\right)}-1.$$ (9)

The goal, of course, is for each robot i to minimize AEI(i,s) (henceforth, AEI for short) as it is an *aligned* reward function, to be used in a reinforcement learning algorithm. Robots minimizing it will necessarily minimize also the swarm’s $EI_{\mathrm{tot}}$ and, thus, the swarm’s CO (Theorem 2). This will maximize the swarm’s utility, as shown in Theorem 1. The assumptions made in the development of the model are summarized in Table 1. We remind the reader that a nomenclature is given in Table A1. In practice, to minimize AEI(i,s) , the robot can attempt to minimize $A_{i}\left(s\right)$ and/or improve $P_{i}\left(s\right)$ . Minimizing the counterfactual $A_{i}^{\phi }\left(s\right)$ requires the robots to estimate the impact of the agent on others, as detailed in the next section.

**TABLE 1:** Assumptions made in the development of the theoretical model and the motivation for creating them.

Assumption	Motivation
Gains in avoidance time are zero	Robots cannot directly contribute to the task when they focus on conflict resolution and avoid collisions
Gains are proportional to program time	The more a robot works uninterrupted, the higher its gains will be; assumption for theoretical derivation
Costs are proportional to time	Robots spend resources (e.g., power) when operating. Longer operations lead to more spending; assumption for theoretical derivation
Outcomes of robots’ actions do not depend on the history of method choices	Without learning, the success of collision avoidance in past collisions does not impact its success in the current collision; for theoretical derivation
Cycle length is the same for all robots for a joint action	The theoretical model is synchronous, for all N agents
When a robot is hypothetically absent, its gains are zero	By definition, it cannot contribute

## 4 Swarming in practice, through learning

We now turn to using the derived reward AEI in practice. Having no ability by the robot to estimate the cycle length when it is hypothetically absent, there are several gaps between the theory and practice: (i) computing AEI(i,s) requires knowledge about other robots’ measurements $\left(A_{i}^{\phi }\right)$ ; (ii) collisions in practice are not necessarily synchronous, or even mutual; and finally, (iii) avoidance and program times ( A , P ) vary for the same method, breaking the Markov assumption. These gaps are discussed below.

### 4.1 Approximating AEI(i,s)

This practical approximation of AEI(i,s) in Equation 11 is composed of three elements: $A_{i},P_{i}$ (which are internal to the robot, thus known) and $A_{i}^{\phi }$ . The latter requires the robot to know the avoidance times of other robots and predict their change when i is hypothetically absent. This is impractical as the effects of a robot on other robots often impossible to perceive accurately by swarm robots. It is, therefore, necessary to use an estimate $\hat{A_{i}^{\phi }}\left(s\right)$ instead, yielding

$$\hat{AEI\left(i,s\right)}\approx EI\left(i,s\right)+\frac{\hat{A_{i}^{\phi }}\left(s\right)}{A_{i}\left(s\right)+P_{i}\left(s\right)},$$

where $\hat{A_{i}^{\phi }}\left(s\right)\approx \sum_{j\in N\backslash \left\{i\right\}}\left[A_{j}\left(s_{i},s_{-i}\right)-A_{j}\left(\phi_{i},s_{-i}\right)\right]$ .

As a first step, we impose a structure on the approximation, setting $\hat{A_{i}^{\phi }}\left(s\right)≔n_{a}\cdot A_{0}$ , where $n_{a}$ is the number of robots affected by this robot and $A_{0}$ is an approximation of how much avoidance time was added or removed to each robot due to the presence of robot i . One way of measuring $n_{a}$ is by the number of robots in the vicinity of the robot i as the collision occurs.

Next, we propose a number of potential values for $A_{0}$ . These will be evaluated empirically (see Section 5 for results). Three immediate estimators are

• $A_{0}=0$ . Setting $A_{0}=0$ yields $\hat{AEI\left(i,s\right)}=EI\left(i,s\right)$ , demonstrating that EI is a special case of AEI , where the avoidance times of other robots are unaffected.
• *Same for all*, $A_{0}=A_{i}\left(s\right)$ . This assumes each robot’s change to avoidance time is worsened, by $A_{i}\left(s\right)$ .
• *Average over time*, $A_{0}=\frac{1}{C}\sum_{j=1}^{C}A_{i}\left(s^{j}\right)$ . The addition in avoidance time to other robots is this robot’s average avoidance time measured in its history, for any action.

The last estimate raises the opportunity to utilize the robot’s own experience with the specific action selected as the basis for estimating the effect of the collision on others. Given a history of play $h^{C}$ and a joint action s∈S , we define R(s)⊆{1,…,C} as the subset of collision indices where joint action s was played. In the same manner, we define $R\left(s_{i}\right)$ as the subset of collision indices where the robot i chose individual action $s_{i}\in S_{i}$ , regardless of the actions chosen by other robots. Using this notation, we additionally propose the following possible approximations for $A_{0}$ :

• *Average over actions*, $A_{0}=\frac{1}{\left|S_{i}\right|}\sum_{s^{\prime }\in S_{i}}\frac{1}{\left|R\left(s^{\prime }\right)\right|}\sum_{j\in R\left(s^{\prime }\right)}A_{i}\left(s^{j}\right)$ . The addition in avoidance time to other robots is by measuring this robot’s average avoidance time for each type of method it selected $s^{\prime }\in S_{i}$ and then averaging over those averages.
• *Minimum over actions*, $A_{0}=\min_{s^{\prime }\in S_{i}}\left(\frac{1}{\left|R\left(s^{\prime }\right)\right|}\sum_{j\in R\left(s^{\prime }\right)}A_{i}\left(s^{j}\right)\right)$ . The addition in avoidance time to other robots is by finding the individual action $s^{\prime }\in S_{i}$ that has the lowest average avoidance time.
• *Maximum over actions*, $A_{0}=\max_{s^{\prime }\in S_{i}}\left(\frac{1}{\left|R\left(s^{\prime }\right)\right|}\sum_{j\in R\left(s^{\prime }\right)}A_{i}\left(s^{j}\right)\right)$ . The addition in avoidance time to other robots is by finding the individual action $s^{\prime }\in S_{i}$ that has the highest average avoidance time.

### 4.2 Dealing with asynchronous and non-mutual collisions

An important assumption made in the derivation of the theoretical model is that collisions are synchronous to the swarm: all robots are assumed to be involved in every collision. In reality, as swarms grow in size, collisions between robots are asynchronous and may even be non-mutual (some robots physically involved in a collision may not recognize the collision state).

As it turns out, the effects of breaking this assumption in practice are mild. First, when a collision occurs and a robot cannot recognize it, there is nothing this robot can do but continue in its task, in which case it will not learn from the collision. This is compatible with the expectation that if the robot does not recognize the collision, then its effect on it is negligible. If, however, it does recognize a collision, its learning from it depends only on its own estimates of $\hat{AEI}$ , which do not require any cooperation from the other robot. Thus once again, we expect the learning robot to be able to learn effectively from the collision.

A potential complication in practice may occur, when a robot taking a collision-resolution action may find itself colliding again with the same or other robots. Once again, however, this is addressed easily. Compatibility with the theoretical model is maintained in such cases by preempting the first collision (essentially, treating the entire cycle leading from the first collision to the new collision as a period of collision avoidance, with $A_{i}$ being set to the entire cycle duration). Then, a new collision is declared, with the robot having the opportunity to once again choose a coordination action (s) and learn from its application.

### 4.3 Varying avoidance and program times

An assumption made in theory is that the outcome of a collision, given a joint action selected, remains the same. However, in practice, this assumption breaks from the point of view of the learning robot. First, the robot does not know the joint action played but only its own individual action, which is only a component in the joint action. Thus, as it chooses the same individual action, it may measure different avoidance and program durations, due to other robots varying their own individual actions, synthesizing different joint actions without its knowledge. Second, the cycle length may vary even for the same joint action due to latent environment variables, which states that the robot is unable to sense directly.

To address this, we propose to use an averaging procedure on $A_{i},P_{i}$ and $A_{i}^{\phi }$ and then calculate a $\hat{AEI}$ approximation, which is averaging over a number of collisions $\hat{AEI}=\frac{\bar{A_{i}}+{\bar{A_{i}}}^{\phi }}{\bar{A_{i}}+\bar{P_{i}}}$ . This can cause inaccuracies in learning because the cycle length $A_{i}+P_{i}$ is a real-valued signal in continuous time while sampling of $A_{i},P_{i}$ and $A_{i}^{\phi }$ is discrete.

We treat the learning problem as reinforcement learning in *semi-Markov decision processes (SMDPs)* (Bradtke and Duff, 1994), rather than discrete MDPs. We use the SMDPs to represent discrete sampling of a continuous-time reward and also introduce a Q-Learning variant for SMDPs, called the *continuous-time Q-Learning*. It differs from Q-Learning in the update step: first, the learning rate α is now a function of interval length: the longer the interval, the closer it will be to 1, thus giving more weight to cycles with longer intervals. Second, $A_{i},P_{i}$ and $A_{i}^{\phi }$ are now also scaled, according to the interval length. Algorithm 1 shows the update step. Note that due to the game-theory conventions, s denotes actions, not states, while we use x here to denote the state perceived by the robot. We experiment with this algorithm in comparison with the familiar Q-learning (see next section).

Algorithm 1 Continuous-time Q-Learning.

1:  **procedure** CTQL-UPDATE( $A_{i},P_{i},A_{o},\tau ,\gamma ,x_{i},x_{i}^{\prime },s_{i}$ )
2: $\alpha \leftarrow 1-e^{-\frac{\Delta t}{\tau }}$

3: $A_{i}^{\prime }\leftarrow \left(1-e^{-\frac{A_{i}}{\tau }}\right)$

4: $P_{i}^{\prime }\leftarrow e^{-\frac{A_{i}}{\tau }}\left(1-e^{-\frac{P_{i}}{\tau }}\right)\cdot P_{i}$

5: $A_{i}^{\phi^{\prime }}\leftarrow \left(1-e^{-\frac{A_{i}^{\phi^{\prime }}}{\tau }}\right)\cdot A_{o}$

6: $Q\left(x_{i},s_{i}\right)\leftarrow \left(1-\alpha \right)Q\left(x_{i},s_{i}\right)+\alpha \left(-\frac{A_{i}^{\prime }+A_{i}^{\phi^{\prime }}}{A_{i}^{\prime }+P_{i}^{\prime }}+\gamma \cdot max_{s^{\prime }}\left(Q\left(x_{i}^{\prime },s^{\prime }\right)\right)\right)$

7:  **end procedure**

## 5 Experiments

We report below on experiments that evaluate swarms utilizing the reinforcement learning using the AEI reward function. The results show that these swarms perform *better*, and more so, that these improvements come from the learning, leading to specialization in the robots: they become *heterogeneous* in their reactions to collisions.

Section 5.1 explains the experiment environments (simulation and robots). Then, we present results from experiments utilizing adaption (Section 5.2, and from experiments using learning (Section 5.3). In all sections, we emphasize the role of heterogeneity.

### 5.1 Experimentation environments

We conducted experiments in two environments: the *Alphabet Soup* order picking simulator (Hazard and Wurman, 2006) created by Kiva Systems engineers, and the Krembot swarm robots, built by Robotican^3^. Videos showing the physical in simulated robots and an overview of the evolution of the EI reward are available online on the project’s web page^4^.

**FIGURE 4:**
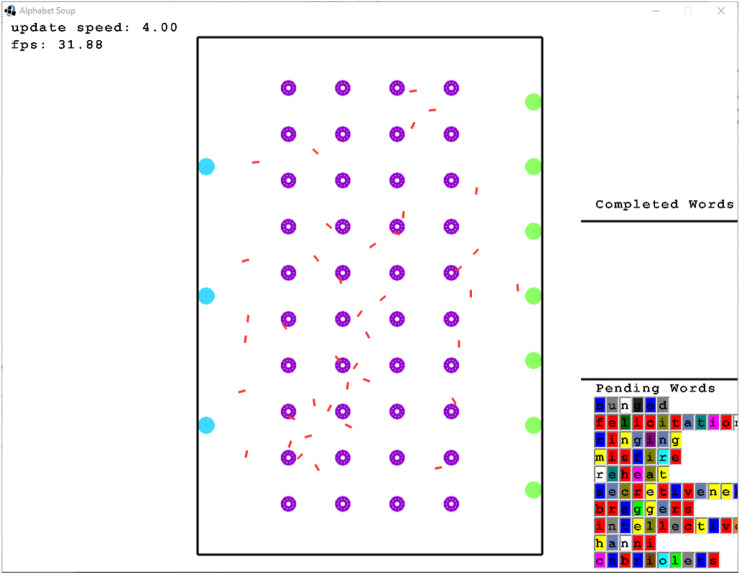
Alphabet Soup simulator. Red lines are the robots, purple circles are the buckets, green circles are the word stations, and cyan circles are the letter stations.

The Alphabet Soup simulator simulates 2D continuous-area order picking by considering the items as letters and orders (combinations of items) as words. Several *word stations* are positioned in the area, each with a list of words to be composed. *Buckets* which contain letters, *letter stations* that are used to re-fill buckets with letters and robots. The robots have three main tasks: the first is to take a bucket to a word station in order to put one letter in this station. The second is to return a bucket to its original position, and the third is to take a bucket to a letter station. Figure 4 shows a screenshot of the simulator in action.

The simulator comes with a centralized task allocation mechanism, which we do not modify. The original collision avoidance mechanism in place is run individually by each robot. It is a reactive heuristic which is a combination of dynamic window (moving towards most vacant direction) and waiting in place for a random amount of time. This mechanism was replaced by an algorithm-selection mechanism, which can choose between various reactive collision-avoidance algorithms, including the original. This choice would be governed by a learning algorithm (as described above) or a different method.

The main measurement of performance for this simulator is the amount of letters placed in word stations in a given amount of time. Unless stated otherwise, each simulation is 10 min long with the last 30 s used for measuring performance and other statistics.

The Krembot robots were used in a variant of multi-robot foraging, where the objective of the robots is to find as many items in a given time. They have relatively limited sensing and processing capabilities. They are cylindrical-shaped robots with a height of 10.5 cm and a diameter of 6.5 cm. Despite their limited sensing capabilities, those robots can detect collisions and also distinguish between a robot and a static object.

The behavior of the robot was controlled by three behavioral states and a few transitions, triggered by specific perceived events. The three states are as follows:

• *Wander*: Search for a station by randomly wandering over the field. Whenever the robot is in this state, its LED light will be magenta (both red and blue simultaneously).
• *Go to homebase*: Go to the homebase to retrieve the item after a station was found. When the robot is in this state, its LED light will be blue.
• *Resolve conflict*: The robot enters this state when it detects an imminent collision with another robot (not a static obstacle). In this state, the robot learns and chooses a reactive coordination method. When the robot is in this state, its LED light will be red.

If a robot detects an imminent collision with a static obstacle, it executes a fixed behavior, unlike with a robot where it executes a coordination method by reactive method arbitration. For each of the three states, there are several transitions from it to other states:

• *Wander* → *Go to homebase*: The robot found a station.
• *Go to homebase* → *Wander*: The robot retrieved an item to the homebase.
• *Wander/Go to homebase* → *Resolve Conflict*: The robot detected an imminent collision with another robot.
• *Resolve Conflict* → *Wander/Go to homebase*: The robot finished executing the reactive coordination method and goes back to its previous state.

**FIGURE 5:**
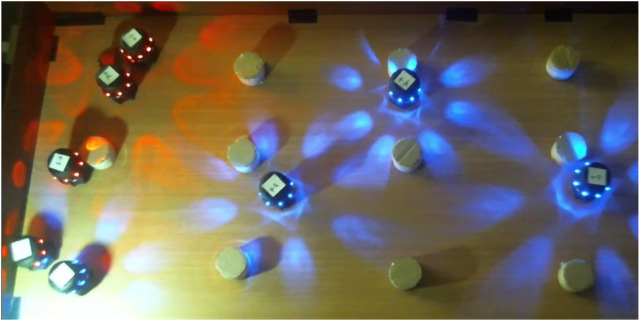
Krembot swarm robots.

Figure 5 shows the environment where experiments with the Krembots were conducted. On the table, the wooden cylinders are the stations where robots gather items from. The arena consisted of a 150×80 ${\text{cm}}^{2}$ tabletop, where we evenly spread 11 stations, fixed in position. Once a robot reaches one of those stations, an item is considered taken, and the robot needs to simulate transporting it, by moving back to the a small area devoted to be a home. It should be noted that since the Krembot robots have no localization capabilities, they are unable to either remember or plan a path to one of the stations not home. Therefore, they do it by randomly searching for a station. The home is lighted (bottom left corner in Figure 5) for identification.

### 5.2 Heterogeneity in adaptation

We distinguish between *learning* and *adaptation*. Learning focuses on *converging to a policy* which consistently chooses the best action for each state. On the other hand, adaptation focuses on *rapidly changing between policies*, according to what is best now.

Previous work by Kaminka et al. (2010) focused on adaptation. To do this, they used stateless Q-Learning with a very high learning rate (as high as 0.8). This allows the robots to rapidly switch between policies; the robots do not typically converge to a particular preferred choice. The reward function used was the original EI (which is a special case of the $\hat{AEI}$ we discuss).

We began by evaluating the use of EI -based adaptation (learning rate α=0.5 ) versus convergent learning (α≤0.1) , in the two environments: Alphabet Soup and Krembot foraging. The goal is to examine whether heterogeneous swarms do better and whether adaptation or learning leads to performance increases.

#### 5.2.1 Adaptation is better in Alphabet Soup

As a first step, we briefly summarize early results evaluating the use of EI -based adaptation in the Alphabet Soup simulator, published by Douchan and Kaminka (2016). They tested the performance of five reactive methods alone (i.e., each used by a homogeneous swarm, where all robots use the same collision avoidance method). Three of these have been used by Rosenfeld et al. (2008) and Kaminka et al. (2010): *Repel* [moving back for 20 ms, as introduced by Rosenfeld et al. (2008)], *Noise* [moving toward a random direction for 20 ms, as introduced by Balch and Arkin (1998)], and *Aggression* [randomly choose between backing off like in repel, or staying put until the robot has moved, as introduced by Vaughan et al. (2000)]. Two additional methods were provided by the Alphabet Soup simulator: *Best Evade* (always go to most vacant direction for a given amount of time) and the default method (termed *Original*), a stochastic combination of *best evade* and noise.

Douchan and Kaminka (2016) compared the performance of these five basic methods with random selection of methods by each robot, in each collision (a *Random* selection method), and with an adaptive use of stateless Q-learning (learning rate α=0.5 and exploration rate ϵ=0.1 ). All settings tested in group sizes vary between 10 and 40 and were repeated 60 times.

**FIGURE 6:**
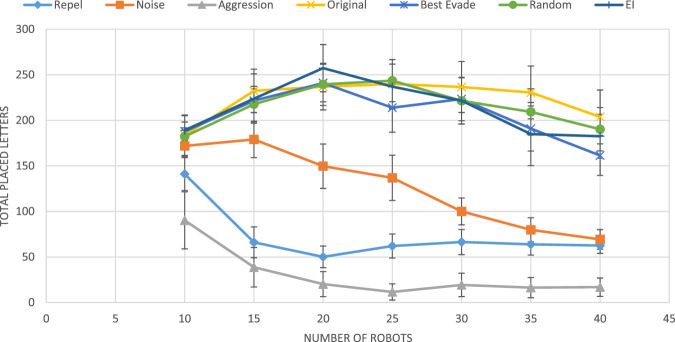
Results obtained by Douchan and Kaminka (2016). The horizontal axis marks the group size, and the vertical axis represents the group performance in terms of total placed letters.

Figure 6 shows the results from these experiments. The figure shows that three of the fixed collision avoidance methods (repel, noise, and aggression) are inferior to the others. These three are behaviorally homogeneous swarms: all robots use the same collision-avoidance methods. Of the four top performers (not necessarily statistically distinguishable), three are behaviorally heterogeneous: the *Random* method, by definition, has every robot change its selected collision-avoidance method with every collision, independently of other robots; the *Original* method stochastically switched between *best-evade* and *noise*; the *EI* method is the adaptive method using the original EI as a reward function, i.e., AEI with $A_{0}=0$ .

Intrigued by these results, we used the Alphabet simulator to directly evaluate the level of heterogeneity of the swarm and its effect on performance, especially in relation to the use of the EI reward. Fixing the group size to 20, we focused on the only homogeneous method that proved to perform well in the experiments reported above: *best evade*. We allowed each robot to select between two variants of this method: *Best evade* for 20 ms (*BE20*) and *best evade* for 2000 ms (*BE 2000*).

We then evaluated four configurations of the robots selections: in the *individual mix* configuration, each of the robots, when entering a collision, chooses BE20 with probability p/100 and BE2000 with probability (1−p)/100 , independently of others. These robots make heterogeneous choice that vary over time. In the *population mix* configuration, p percent of the robots always chooses BE20, and the rest always chooses BE 2000; their choices do not vary with time. These configurations allow systematic evaluation of the level of heterogeneity: when p=0 , the swarm is homogeneous, and all robots select BE 2000; when p=100 , all robots use BE20. In between these two extremes, the swarm is heterogeneous, more-so in the individual mix configuration than in the population mix. We emphasize these are not learning methods: p is controlled and fixed.

**FIGURE 7:**
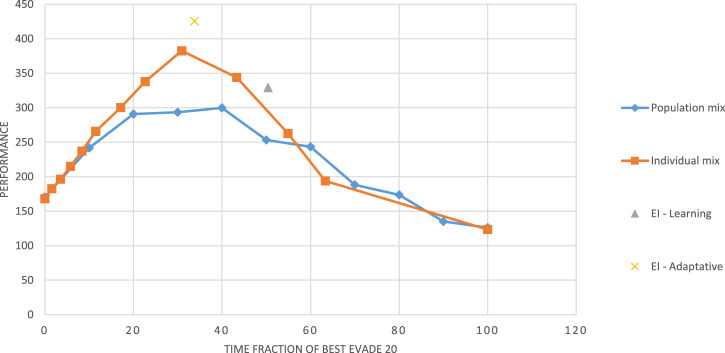
Swarm performance as a function of the heterogeneity of the swarm (time fraction of BE20, p ).

Figure 7 shows the performance of the two configurations as a function of the fraction of BE20 in Alphabet Soup as p is controlled and varied between 0 and 100. We interpolate linearly between the sampled experiment points. The figure shows that both controlled-mix configurations reach their maxima points at p between approximately 30 and 50, i.e., where only between 30% and 50% of the robots select BE20. Both homogeneous swarms shown (at fraction = 0 and at fraction = 100) have the lowest performance.

The figure also shows two specific performance points, resulting from the application of reinforcement learning with the EI reward. The *EI-Adaptation* point marks the result of using learning with a learning rate α=0.5 and exploration rate of 0.1, both encouraging rapid changes in the learned policy, just as the individual mix changes selection by the same robot over time. The *EI-Learning* point marks the result of using learning rate α=0.05 and exploration rate of 0.02, to encourage convergence to a single selection (just as the population mix enforces). We note that the adaptive method outperforms the individual mix and the learning method outperforms the population mix, and both points are reached when the swarm is behaviorally heterogeneous.

#### 5.2.2 Adaptation is not always better in Krembots

We now turn to testing the role of adaptation and learning with real robots, hoping to draw lessons as to the role of heterogeneity in these different settings. We test two coordination methods of the same type but with different time parameters (the speed of the robots is different, and so these were empirically determined): Best Evade for 500 ms (*BE500*) and Best Evade for 10,000 ms (*BE10000*). We first test each method separately and then perform test selection using *EI-Adaptation* (learning rate α=0.5 and an exploration rate of 0.1) and *EI-learning* (learning rate α=0.05 and an exploration rate of 0.02).

We tested the performance of the different configurations in four robots and eight robots. We measure the performance of each configuration and the time fraction the robots spent on *BE500*. The duration of each run is 1 h long. For each hour-long run, we logged each event, such as a collision or an item that was retrieved. From this log, we extracted statistics on the number of items retrieved and the coordination method choices of the robots. We extracted statistics based only on the last 15 min of the run since we want the learning to stabilize. As before, this allows controlling the heterogeneity of the swarm by fixing the fraction of *BE500* or assessing it from the logs.

**FIGURE 8:**
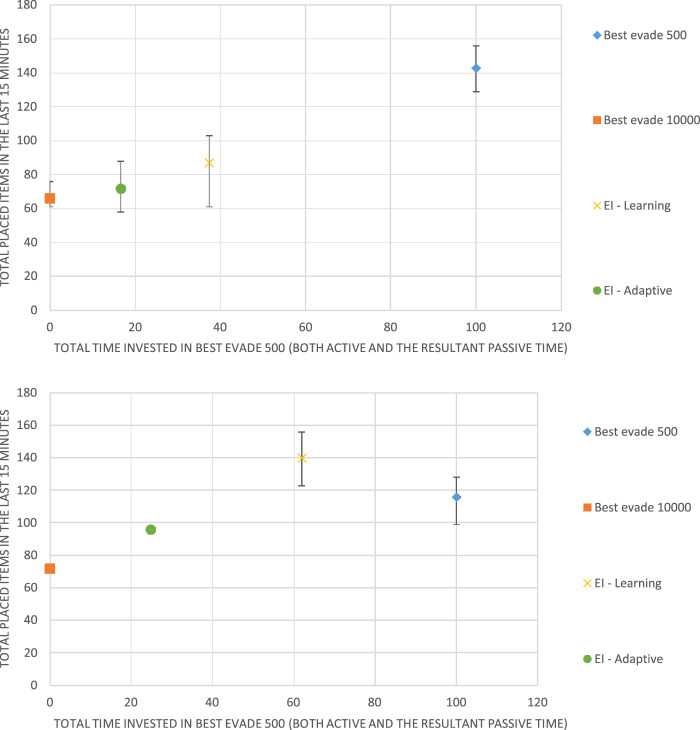
Results for Best Evade 500 (BE500) and Best Evade 10,000 with four (top) and eight (bottom) Krembot robots, respectively. The horizontal axis marks the fraction of BE500 used. The vertical axis marks the group performance in terms of total retrieved items.

Figure 8 shows the results for 4 Krembot robots (top) and for Figure 8 (bottom). Like the previous figure, the horizontal axis measures the fraction of the time, in which the robots spent using BE500, i.e., the behavioral heterogeneity of the swarm: The 0 point on this axis marks a homogeneous swarm that never uses BE500 and instead always uses *BE10000*. The point marked 100 on this axis shows the results for another homogeneous swarm, where all robots use BE500.

Figure 8 shows that in the case of 4 robots (top), the best-performing swarm is a homogeneous swarm (all robots choose BE500 collision-avoidance). Both EI-Adaptive and EI-Learning fail to achieve equivalent performance. However, in the case of 8 robots, a heterogeneous swarm is the best method, and it is achieved using EI-Learning (where about 50% of the robots choose BE500). Here, while heterogeneity proves superior, it is achieved by learning using regular Q-Learning, rather than the adaptive method proposed by Kaminka et al. (2010).

### 5.3 Heterogeneity in learning

As the use of learning does not seem to work stably, we explore it further. In learning, robots converge to a fixed policy. We compare regular Q-Learning to continuous time Q-Learning. We do so by measuring the performance of different WLU approximations (Section 4.1), each with regular Q-Learning and continuous time Q-Learning (Algorithm 1, Section 4.3). The parameters of regular Q-Learning were set as follows: the learning rate was 0.05, and the exploration rate was 0.02. The parameters of continuous time Q-Learning are as follows: τ=10 seconds and the exploration rate was 0.02.

**FIGURE 9:**
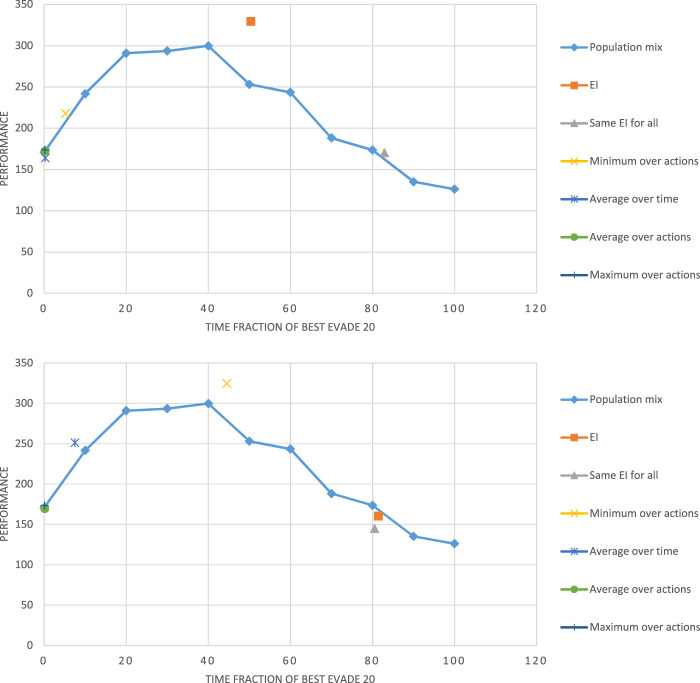
Performance of different WLU approximations with regular Q-learning (upper chart) vs. with continuous time Q-learning (lower chart) and where they are relative to the population mix.

We begin again with the Alphabet Soup simulator, with the action set containing two actions as before: *BE20* and *BE 2000*. Figure 9 shows the results when using regular Q-Learning (top) and continuous-time Q-Learning (bottom). The line shows the population-mix, as before. The top figure shows EI learning being superior to all others (as in Figure 7). The bottom figure shows the *Minimum over actions* being superior. We draw two lessons from these results. First, regardless of the learning method and assumptions, the top performing swarm is always a heterogeneous swarm. Second, the algorithm used is sensitive to the selected $\hat{AEI}$ approximation.

Finally, we go back to the Krembot robots to evaluate the use of the learning algorithms, with different rewards and both adaptive and learning parameters. We tested BE500 and BE10000 with EI (Kaminka et al., 2010) using the same Q-Learning parameters for learning (EI-learning) and adaptation (EI-Adpative). We also evaluate the use of EI with the continuous-time Q-Learning algorithm (Algorithm 1) and, alternatively, the use of the minimum-over-actions approximation with the same algorithm. Its parameters were set to τ=10 seconds and an exploration rate of 0.02.

**FIGURE 10:**
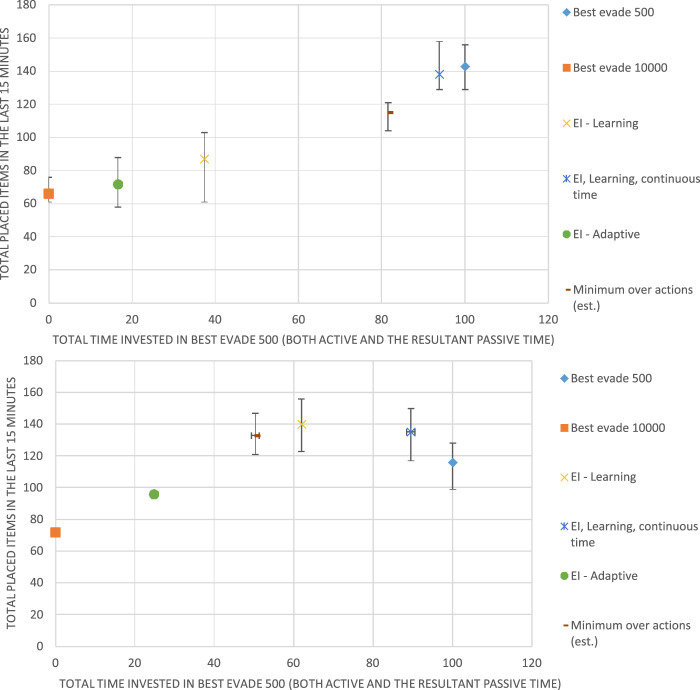
Learning in Krembots for four robots (upper chart) and eight robots (bottom chart).

Figure 10 shows the results. For four robots, as before, a homogeneous swarm (everyone uses BE500) is the best. It is good to see, however, that the use of Algorithm 1 with EI comes very close to its performance. Indeed, it results in a heterogeneous swarm where 95% of robots select BE500. Given the exploration rate and the fact that there are only two methods, this corresponds to exactly the 5% of the time where the exploration rate forces the robot to choose BE10000. The bottom figure shows that all best swarms are heterogeneous.

In a different publication, Douchan et al. (2019) reported on additional experiments utilizing the learning methods we presented, contrasting their results with those achieved by testing directly with the true swarm utility U , WLU of the utility U , and several WLU alternatives. We refer the reader there, for further details.

## 6 Conclusion

This paper explores the role of behavioral heterogeneity in robot swarms engaged in foraging. It presents an abstract theoretical model of this swarm task, showing a mathematical connection between the *Coordination Overhead* (CO) of the robots in foraging—defined by the portion of time spent coordinating—to the global utility of the swarm. We then connected between the swarm CO of the whole lifetime of the swarm, to the decisions of individual robots in a single collision. This allows us to show that in principle, swarm robots can maximize an individual reward for each collision that will yield good global utility in the long run.

Specifically, we presented the *Aligned Effectiveness Index* (AEI) , a reward function that ties the global CO of the swarm with individual estimates. This reward function allows individuals within the swarm to make decisions that improve the swarm’s performance while adapting to changing collision conditions. It is a generalization of the EI reward proposed in earlier work Kaminka et al. (2010), which is not aligned, and for which the bounds we present are not known.

We focused on *swarm foraging*, a canonical swarm task of great interest both scientifically and commercially (e,g., in order picking, search and rescue, and agriculture; see applications discussed in Section 2). We have shown several solutions to challenges that may rise in practice when applying the theoretical model. First, we discussed several possible approximations for the AEI reward function that stands at the basis of utilizing learning in this task. Second, we developed a continuous time variant of Q-Learning in order to address possible inaccuracies of regular Q-Learning that may rise in continuous-time settings, in which robots operate. The utilization of learning by agents, in a completely distributed manner, often leads to specialization of behavioral roles, and thus to more heterogeneous swarms.

The results of the experiments clearly support the hypothesis that *diversity in decision-making* can play an important role in the performance of a swarm. This conclusion agrees with studies of swarms, whose members evolve their decision-making controllers using evolutionary computation (Montague et al., 2023), and studies of behavioral diversity in models of human pedestrians [e.g., in mixed culture (Kaminka and Fridman, 2018)]. Surprisingly, perhaps, Balch (1999) has investigated the use of machine learning in simulated foraging robots (up to 8) and reached conclusions opposite from ours, which states that foraging robots seemed to benefit from being homogeneous. We believe that this seeming contradiction in conclusions is a result of the previous study utilizing robots that were able to communicate information about the location of items and home. We also note that the results demonstrate that diversity *can* be extremely important to the success of the swarm but is not always needed. For instance, in the experiments we conducted, homogeneous decision-making seems to do well in smaller swarm sizes (see Figure 10).

The conclusion we reach on behavioral diversity complements analogous conclusions as to the importance of *diversity in capabilities*, in related studies. For example, the *swarmanoid* project (Dorigo et al., 2012) explores the use of mechanically different robots in carrying out complex tasks. Berumen et al. (2023) demonstrated the impact of robots with diverse error models on foraging performance, and Adams et al. (2023) developed foraging swarms made of two types of robots: *searchers* and *beacons* that assist in communicating signals in-limited communication settings. Swarms of heterogeneous nanobots are able to carry out complex tasks, e.g., a form of Asimov’s laws of robots (Kaminka et al., 2017). In the larger context of multi-robot systems, similar ideas about the importance of diversity have been presented in investigations of heterogeneous *teams*, where robots communicate globally and essentially without restrictions so as to coordinate how to bring their different capabilities to bear on the joint problem. Such investigations include those by Xu et al. (2005), Parker and Tang (2006), Tang and Parker (2007), and Liemhetcharat and Veloso (2013). Likewise, heterogeneity plays an important role in natural swarms as well, e.g., see (Ariel et al., 2022).

Although this study focused on *diversity* of the swarm, complementary studies examine the *optimality* of the individual robot’s decision-making, when robots use aligned rewards. Recent investigations of alternative learning approaches and alternative formulations begin to explore the question of how individual self-interested rational reward maximization leads to collective utility maximization (Fatima et al., 2024; Katz, 2023; Kaminka, 2025).

## Data Availability

The raw data supporting the conclusions of this article will be made available by the authors, without undue reservation.

## Footnotes

1 Human workers used to walk 20 km a day to pick ordered items in shelves in such warehouses, and labor was in short supply.

2 The definition of coordination in Danassis and Faltings (2018) differs from the definition of coordination in our work. We define coordination as the need to take an action due to an interaction between agents. They define coordination as a *consensus*: where agents need to choose the same action in order to achieve optimal results.

3
https://www.robotican.net/kremebot

4
https://www.cs.biu.ac.il/∼galk/research/swarms/

## Appendix A: Nomenclature

**TABLE A1:** List of symbols and notations used in this paper. Where appropriate, we also list equivalent notation used by Kaminka et al. (2010).

Symbol	Meaning	Equivalent in Kaminka et al. (2010)
N={1,2,…,n}	Set of n=|N| robots (players)	$A=\left\{a_{1},\ldots ,a_{n}\right\}$
$S_{i}$	Set of actions available for robot i	M
$s_{i}\in S_{i}$	Specific action taken by robot i	$\alpha_{i}$ or α
$S=S_{1}\times \cdots \times S_{n}$	Set of all possible joint actions	
$s=\left(s_{1},\ldots ,s_{n}\right)\in S$	Joint action, a combination of the individual specific actions of all robots	
$s_{i}^{j}$	Specific action of the robot i , in collision j	
$s^{j}$	Joint action taken in collision j	
$h^{j}=\left(s^{1},s^{2},\ldots ,s^{j}\right)$	History of joint actions played up until (and including) collision j	
$S^{j}$	Set of all possible joint action histories until (and including) collision j	
$g_{i}:S^{j}\mapsto R$	Gain by robot i as a function of the joint actions played up until (and including) collision j	gain
$c_{i}:S^{j}\mapsto R$	Cost incurred by robot i as a function of the joint actions played up until (and including) collision j	$C_{i}^{C},c$
$u_{i}:S^{j}\mapsto R$	Utility of player i , given the joint actions played up until (and including) collision j	$u_{i}\left(\alpha_{i}\right)$
$U≔{⋃}_{i\in N}u_{i}$	Set of utilities of each player as a function of the joint actions played up until (and including) collision j	
$A_{i}:S^{j}\mapsto R$	Active time of the player i as a function of the joint actions played up until (and including) collision j	$I_{i}^{a}$
$P_{i}:S^{j}\mapsto R$	Passive time of the player i as a function of the joint actions played up until (and including) collision j	$I_{i}^{p}$
$l_{i}\left(s\right)$	Cycle length of robot i , given joint action s : $A_{i}\left(s\right)+P_{i}\left(s\right)$	
$EI\left(i,s\right)≔\frac{A_{i}\left(s\right)}{A_{i}\left(s\right)+P_{i}\left(s\right)}$	Effectiveness index (Kaminka et al., 2010)	$EI_{i}\left(s\right)$
$EI_{\mathrm{tot}}\left(s\right)$	Sum of $EI_{i}\left(s\right)$ over all agents i∈N	
AEI(i,s)	Aligned effectiveness index, defined as the difference reward (WLU) with respect to global function $EI_{\mathrm{tot}}$ , i.e., $\Delta_{i}^{EI_{\mathrm{tot}}}\left(s\right)$	
C∈N	Number of collisions during swarm lifetime	
T∈R	Total swarm lifetime duration	T
